# Designing for usability: development and evaluation of a portable minimally-actuated haptic hand and forearm trainer for unsupervised stroke rehabilitation

**DOI:** 10.3389/fnbot.2024.1351700

**Published:** 2024-04-04

**Authors:** Raphael Rätz, Alexandre L. Ratschat, Nerea Cividanes-Garcia, Gerard M. Ribbers, Laura Marchal-Crespo

**Affiliations:** ^1^Motor Learning and Neurorehabilitation Laboratory, ARTORG Center for Biomedical Engineering Research, University of Bern, Bern, Switzerland; ^2^Department of Cognitive Robotics, Delft University of Technology, Delft, Netherlands; ^3^Department of Rehabilitation Medicine, Erasmus MC, University Medical Center Rotterdam, Rotterdam, Netherlands; ^4^Rijndam Rehabilitation Center, Rotterdam, Netherlands

**Keywords:** neurorehabilitation, robotic, home rehabilitation, group therapy, haptic rendering, portable, grasping, usability

## Abstract

In stroke rehabilitation, simple robotic devices hold the potential to increase the training dosage in group therapies and to enable continued therapy at home after hospital discharge. However, we identified a lack of portable and cost-effective devices that not only focus on improving motor functions but also address sensory deficits. Thus, we designed a minimally-actuated hand training device that incorporates active grasping movements and passive pronosupination, complemented by a rehabilitative game with meaningful haptic feedback. Following a human-centered design approach, we conducted a usability study with 13 healthy participants, including three therapists. In a simulated unsupervised environment, the naive participants had to set up and use the device based on written instructions. Our mixed-methods approach included quantitative data from performance metrics, standardized questionnaires, and eye tracking, alongside qualitative feedback from semi-structured interviews. The study results highlighted the device's overall ease of setup and use, as well as its realistic haptic feedback. The eye-tracking analysis further suggested that participants felt safe during usage. Moreover, the study provided crucial insights for future improvements such as a more intuitive and comfortable wrist fixation, more natural pronosupination movements, and easier-to-follow instructions. Our research underscores the importance of continuous testing in the development process and offers significant contributions to the design of user-friendly, unsupervised neurorehabilitation technologies to improve sensorimotor stroke rehabilitation.

## 1 Introduction

Stroke is one of the main contributors to disability worldwide and its impact on society is expected to further increase in the future with aging populations (Feigin et al., 2022). After stroke, the loss of upper-limb functions such as grasping and fine manipulation is particularly prevalent (Lai et al., 2002; Kwakkel et al., 2003; Zbytniewska-Mégret et al., 2023) and affects the autonomy and quality of life of patients (Mercier et al., 2001).

To maximize therapy outcomes, patients should undergo an intense and high-dosage (i.e., large number of repetitions and long overall training duration) neurorehabilitation program (Kwakkel et al., 2004; Schneider et al., 2016; Ward et al., 2019; Tollár et al., 2021). Unfortunately, the dosage and intensity of current interventions that rely on one-to-one interactions between therapists and patients are often considerably lower than recommended due to organizational constraints and limited resources in the healthcare system (Camillieri, 2019). This situation is expected to be further aggravated in the near future by the increasing financial pressure in healthcare and the global clinical staff shortage (Haakenstad et al., 2022). Group therapy and home rehabilitation are two approaches that could mitigate this societal challenge. Group therapy can be as effective as dose-matched individual therapy (Renner et al., 2016), while home-based rehabilitation provides the flexibility of location and can maintain therapy dosage following clinical discharge if exercises are frequently performed according to plan and executed correctly (Hackett et al., 2002; Cramer et al., 2019; Chi et al., 2020). It has been suggested that sustained training at home might even be a prerequisite for minimizing subsequent losses in patients' quality of life (Tollár et al., 2023). Thus, a paradigm shift is needed from conventional on-site labor-intensive motor rehabilitation to minimally supervised motor rehabilitation at the patient's home.

However, both group therapy and at-home rehabilitation require adequate tools that deliver high-dosage training and ensure patient engagement in minimally supervised (group therapy) or unsupervised environments (home rehabilitation). Sensorized or robotic devices in combination with interactive gamified exercises are promising candidates to drive this paradigm shift (Chen et al., 2019; Handelzalts et al., 2021; Lambercy et al., 2021; Forbrigger et al., 2023a). The effectiveness of robot-based group therapy in providing high dosage high-intensity therapy has already been demonstrated (e.g., Hesse et al., 2014), while the feasibility of sensorized or robotic devices to deliver high dosage in self-guided therapies at home has been largely endorsed, (e.g., Sivan et al., 2014; Wittmann et al., 2016; Hyakutake et al., 2019; McCabe et al., 2019; Rozevink et al., 2021). Notably, there is even evidence that technology-based rehabilitation programs can outperform conventional (i.e., non-interactive exercises according to paper instructions) home rehabilitation (Wilson et al., 2021; Swanson et al., 2023).

Among current technological solutions for unsupervised home rehabilitation, we can find non-actuated devices like the Armeo^®^Senso (Hocoma AG, Switzerland), the FitMi (Flint Rehab, USA) or MERLIN (Guillén-Climent et al., 2021). These sensorized devices typically track the patient's movements using sensors such as inertial measurement units (IMUs) or sensorized wheels (e.g., rotary encoders), allowing the patients' movements to be used as inputs for gamified exercises on a tablet, computer, or smartphone. Some devices, like the Gripable (GripAble Limited, United Kingdom), Pablo^®^ (TyroMotion GmbH, Austria), or the NeuroBall^TM^ (Neurofenix, USA) additionally feature sensors to detect grip strength. While non-actuated devices have shown their feasibility to deliver high dosage in self-guided therapies at home (Wittmann et al., 2016; Rozevink et al., 2021), they are limited in their capabilities to actively support or resist patients' hand movements. This is overcome with actuated robotic devices such as the PoRi, a compact hand-held device with one actuated degree of freedom (DoF) for grasping that includes haptic feedback vibro-tactile actuators (Wolf et al., 2022). Other examples include the hCAAR, a robotic device for planar movements in the transversal plane (Sivan et al., 2014), or the Motus Hand, a commercial device for wrist flexion/extension (Wolf et al., 2015). Yet, while all these solutions seemed to be well suited for minimally supervised or unsupervised training, except for PoRi, they mostly target motor functions and neglect the training of somatosensory functions.

The execution of skillful movements relies on the integration of meaningful sensory information such as touch and proprioception (Scott, 2004; Pettypiece et al., 2010) and the provision of such information during training is therefore highly recommended (Bolognini et al., 2016; Handelzalts et al., 2021). In robotic training, such sensory information can be provided through haptic rendering—i.e., the generation of physical forces from interactions with tangible virtual objects (Gassert and Dietz, 2018). Although multiple robotic rehabilitation devices have specifically been developed to address this (e.g., Metzger et al. 2011; Fong et al. 2017; Rätz et al. 2021a), they are mostly intended for clinical rehabilitation. To our best knowledge, the recent ReHandyBot (Articares Pte Ltd, Singapore)—a commercial device based on the haptic tabletop device HandyBot (Ranzani et al., 2023) with two DoF, i.e., grasping and pronosupination—is currently the only commercial portable upper-limb device intended for home use which was explicitly designed to also address sensory deficits.

Commercial devices also remain costly, limiting their adoption both for group therapy and at-home rehabilitation. Cost-effectiveness was listed as one of the driving reasons for not recommending robot-assisted neurorehabilitation in adult post-stroke training by the United Kingdom National Institute for Health and Care Excellence guidelines in October 2023 (NICE, 2023). These costs were not only associated with the device purchase (first investment) but also with maintaining the equipment, the staff time for setting up the machine for each use, and time to teach the patient how to use it. Further, it was noted that machines were only used in a small subset of patients and so could not be used at their full capacity, increasing the cost per use and so overall intervention costs. We thus think that there is a further need for low-cost, versatile, intuitive, and highly portable hand rehabilitation devices that provide meaningful haptic feedback for use in minimally supervised or unsupervised settings.

Here, we present the design and results from a first usability test of our second prototype of a portable and low-cost haptic hand trainer based on a novel compliant shell mechanism. The device offers two degrees of freedom: An actuated one for grasping as well as haptic rendering, and a passive one for pronosupination movements. Meeting the stringent criteria for home rehabilitation devices is challenging (Chen et al., 2019; Forbrigger et al., 2023b). It has been shown that usability and users' perceptions of assistive devices and rehabilitation technology for home use substantially influence their long-term utilization (Biddiss and Chau, 2007; Sivan et al., 2014; Sugawara et al., 2018; Ciortea et al., 2021). Therefore, we co-created the novel portable device with clinical personnel following a human-centered design approach to ensure efficient and goal-oriented development. For this purpose, we followed four phases when designing our solution: (i) Understand the context of use; (ii) Specify patients' requirements; (iii) Design the solution; and (iv) Evaluate against requirements. Insights from the first two phases are published in Rätz et al. (2021b) and Van Damme et al. (2022). Here we report on the two later phases. We embraced a mixed-method approach to evaluate the usability of our invention, where quantitative methods such as questionnaires were combined with qualitative approaches like interviews. Hereby, the use of standardized questionnaires was advocated as the results become more meaningful and comparable (Meyer et al., 2021). We included supplementary techniques like video recordings and eye-tracking to help gain further insights into the root causes of usability issues (Goldberg and Wichansky, 2003; Schaarup et al., 2015; Maramba et al., 2019). For a comprehensive online guide on usability assessment methods, see Meyer et al. (2023).

The rest of the paper is organized as follows: We first present the development of the second prototype of the novel portable hand trainer as well as the design of an accompanying rehabilitation game including the computation of interaction forces with virtual game objects. This development is the continuation of a concept that we proposed in Van Damme et al. (2022). We then introduce the setup and methodology of a usability experiment with 13 healthy participants, including three therapists, in a simulated unsupervised scenario. Finally, we present the results and discuss their implications for further developments and studies in patients' homes. This paper evaluates our design choices and offers other device and rehabilitation game developers detailed insights into our learnings.

## 2 Materials and methods

### 2.1 Device development

#### 2.1.1 Requirements

The first prototype of our portable hand trainer was developed and patented in 2022 (Van Damme et al., 2022; Rätz et al., 2023). The core idea of this first prototype was a U-shaped compliant shell that is grasped with the entire hand—i.e., enclosed with fingers, thumb and palm—and could allow an extremely simple and inherently safe mechanical human-device interaction even in case of improper setup of the user's hand. This shell design mimics a natural large-diameter power grasp, i.e., simultaneous flexion or extension of all fingers with abducted thumb. This particular grasp was selected as it represents one of the most frequently employed hand movements in activities of daily living (ADL) (Bullock et al., 2013) and is effectively trained in clinical rehabilitation (Pandian et al., 2012). Importantly, our first prototype drastically minimized the risk of skin getting pinched in gaps between moving parts regardless of the exact hand proportions of the user, making it an excellent candidate for home rehabilitation. Finally, it also featured a highly-backdrivable transmission and offered good mechanical transparency, allowing for open-loop impedance control to achieve fine haptic rendering. While the initial prototype served as a preliminary proof of concept, a more elaborate version was clearly needed for a first usability study.

For the second generation of the portable hand trainer presented in this study, we defined the following improvements based on first evaluation tests, informal discussions with therapists of the Department of Neurology, University Hospital Bern, Switzerland, and the literature (e.g., Lu et al., 2011; Akbari et al., 2021; Li et al., 2021; Rätz et al., 2021b): i) The device must be aesthetically pleasing and should look like a medical device. This includes integrating the electronics (except for the power supply and emergency stop) into the device housing. ii) A passive DoF shall be added for pronosupination movements. iii) Although training was feasible for various hand sizes with the first prototype, we found that shorter fingers would benefit from smaller shell sizes. In addition, we also aimed to increase the range of motion of fingers and thumb during grasping. iv) The device must be safe for the ultimate goal is to make it in real unsupervised training. v) The device should remain as compact and portable as possible.

#### 2.1.2 Shell design

**Figure 1:**
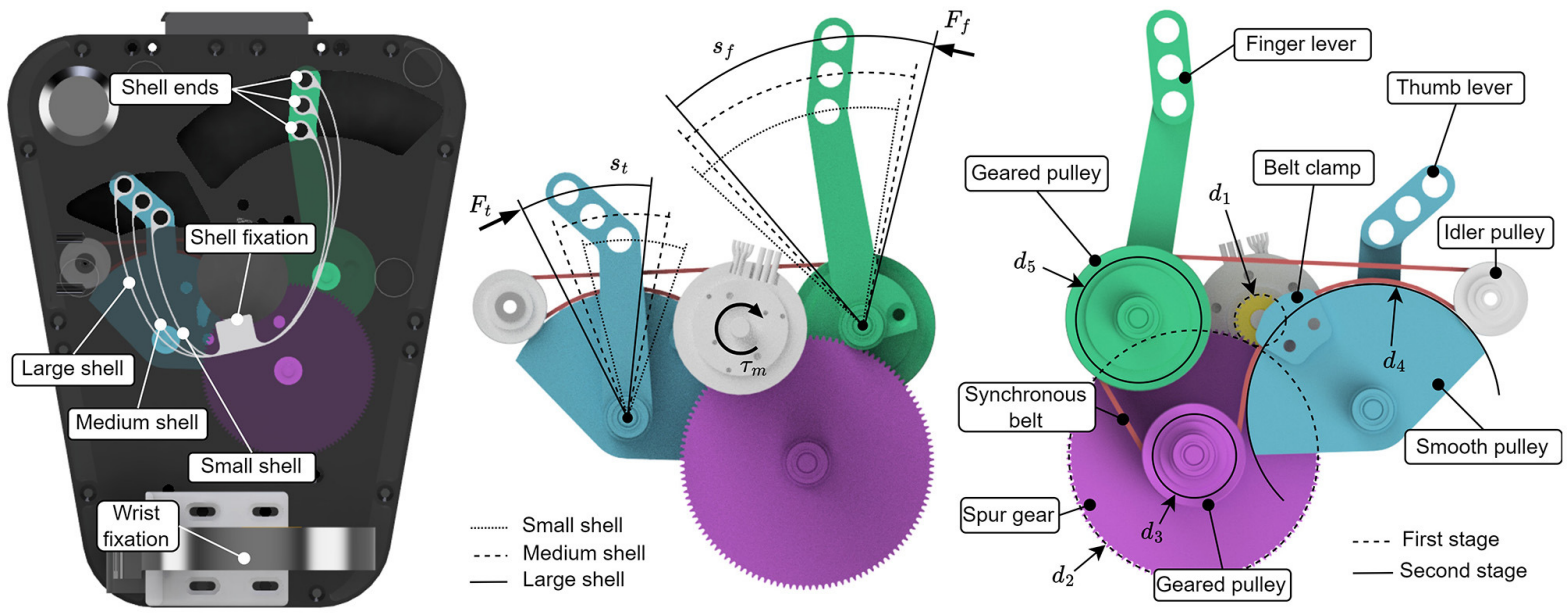
Overview of the shell actuation mechanism. **Left**: Top view of the device and shells with three different sizes. The fixations of the three shells are aligned. The shells are actuated at their ends through levers. **Center**: Top view of the two-stage transmission mechanism with indicated paths of the shell ends. **Right**: Bottom view of the transmission. The thumb and finger pulleys have different diameters, *d*_5_ and *d*_4_ respectively, to account for the different ranges of motion of the thumb and fingers. Note the belt clamp, which allows the use of a smooth pulley (with diameter *d*_4_). The idler pulley is required for the routing of the synchronous belt around the smooth pulley.

The desired bending behavior of the shell during grasping—i.e., following the natural movement of the thumb and the fingers—is achieved by anchoring the shell center part to the device while moving the shell ends on circular paths. For this, the shell ends are connected to a thumb and finger lever (Figure 1). These levers are coupled and actuated through a transmission (see Section 2.1.3).

We decided to design three shell sizes (small, medium, large) for this study's device, using the hand measurements of Garrett (1971). The 5th percentile female hand was considered a small hand, the 95th percentile male hand a large one, and the average of both a medium-sized hand. It is important to note that when closing the hand, the arc length of the inner (i.e., palmar) side of the hand shortens—as easily visible by the skin creases beneath the finger joints—while the thin shell can be assumed to maintain the same arc length when bent. This results in a sliding motion of the fingers along the shell when closing the hand. However, in the first prototype, we found that this sliding is imperceptible to the user and does not pose a problem. In this second prototype, we used this knowledge in the design of three size-specific shell geometries. We designed the size-specific shells such that their ends approximately align with the fingertips (or thumb tip respectively) when the hand is extended (Figure 3). With this and the aforementioned tendency of the fingers to slide backwards on the shell, we know that the fingertips will not collide when the shell is closed. The shell height is the same for the three sizes.

The lengths of the levers that move the shell ends and the locations of their respective center of motion were defined in an iterative process such that (i) the shell ends move along a natural fingertip path, and (ii) there is no collision between mechanical parts. For a quick change of the shells, each shell is mounted with a combination of removable rods at its ends and dowel pins at its center. Thereby, all three shells share a common center fixation, resulting in alignment of the shell center areas that support the thenar web space (i.e., the part between thumb and fingers). This allowed us to use the same wrist fixation for the three shell sizes.

#### 2.1.3 Transmission and control

The device is actuated by an electric DC motor. The transmission is divided into a spur gear stage and a synchronous belt transmission stage. Hereby, the synchronous belt has two functions: First, it amplifies the motor torque, and second, it couples the thumb and the finger movements. Figure 1 shows the mechanism. Note the different diameters of the finger (*d*_5_) and thumb actuation (*d*_4_) pulleys, which allows us to take into account the different ranges of motion of the thumb and the fingers. The arc lengths of the finger and thumb paths are denoted *s*_*f*_ and *s*_*t*_ respectively, and are computed in Eq. (1) with *d*_1_, *d*_2_, *d*_3_, *d*_4_, and *d*_5_ being the effective pulley/gear diameters, *r*_*f*_ and *r*_*t*_ the lever lengths (see Section 2.1.2), and θ_*m*_ being the angular displacement of the motor shaft.


(1a) $$\begin{array}{ll} s_{t} & =\frac{r_{t}}{i_{t}}\theta_{m}\;\text{with }i_{t}=\frac{d_{2}d_{4}}{d_{1}d_{3}} \end{array}$$

(1b) $$\begin{array}{ll} s_{f} & =\frac{r_{f}}{i_{f}}\theta_{m}\;\text{with }i_{f}=\frac{d_{2}d_{5}}{d_{1}d_{3}} \end{array}$$

Applying the principle of virtual work in a static condition, we can thus compute the required motor torque τ_*m*_ with Eq. (2), using the partial derivatives of *s*_*f*_ and *s*_*t*_ with respect to the angular position of the motor shaft θ_*m*_ and the thumb and fingertip forces *F*_*t*_ and *F*_*f*_ (i.e., the forces at the shell ends, along the circular path of the shell ends):


(2) $$\begin{array}{l} \begin{array}{cc} \tau_{m} & =\frac{\partial s_{t}}{\partial \theta_{m}}F_{t}+\frac{\partial s_{f}}{\partial \theta_{m}}F_{f}=\frac{r_{t}}{i_{t}}F_{t}+\frac{r_{f}}{i_{f}}F_{f}. \end{array} \end{array}$$

If we assume that the thumb and finger forces are equal and given by *F* = *F*_*t*_ = *F*_*f*_, the motor torque is given by Eq. (3). Note, that this assumption does not necessarily strictly hold during use, as the additional hand-device contacts at the wrist fixation and thenar web space might result in a statically over-constrained situation where unequal forces to thumb and fingers could be applied. However, this assumption is required for the control of the one-DoF shell actuation.


(3) $$\begin{array}{l} \tau_{m}=r^{\prime }F\;\text{with }r^{\prime }=\frac{r_{t}}{i_{t}}+\frac{r_{f}}{i_{f}} \end{array}$$

Because the thumb and fingertip movements are coupled, we need to compute their combined displacement *s* and the speed ṡ with Eq. (4):


(4a) $$\begin{array}{ll} s & =s_{t}+s_{f}=r^{\prime }\theta_{m} \end{array}$$

(4b) $$\begin{array}{ll} ṡ & ={ṡ}_{t}+{ṡ}_{f}=r^{\prime }{\dot{\theta }}_{m}. \end{array}$$

The desired rendered force *F* is computed with Eq. (5), depending on the desired visco-elastic characteristics (viscosity *B* and stiffness *K*) of the virtual object/environment the participant may interact with.


(5) $$F=\{\begin{array}{ll} K(s-s_{0})+B\dot{s}, & \text{if }s>s_{0}\text{ and }\dot{s}>\text{0}\\ K(s-s_{0}), & \text{if }s>s_{\text{0}}\text{ and }\dot{s}\leq \text{0}\\ 0, & \text{else} \end{array}$$

**Figure 2:**
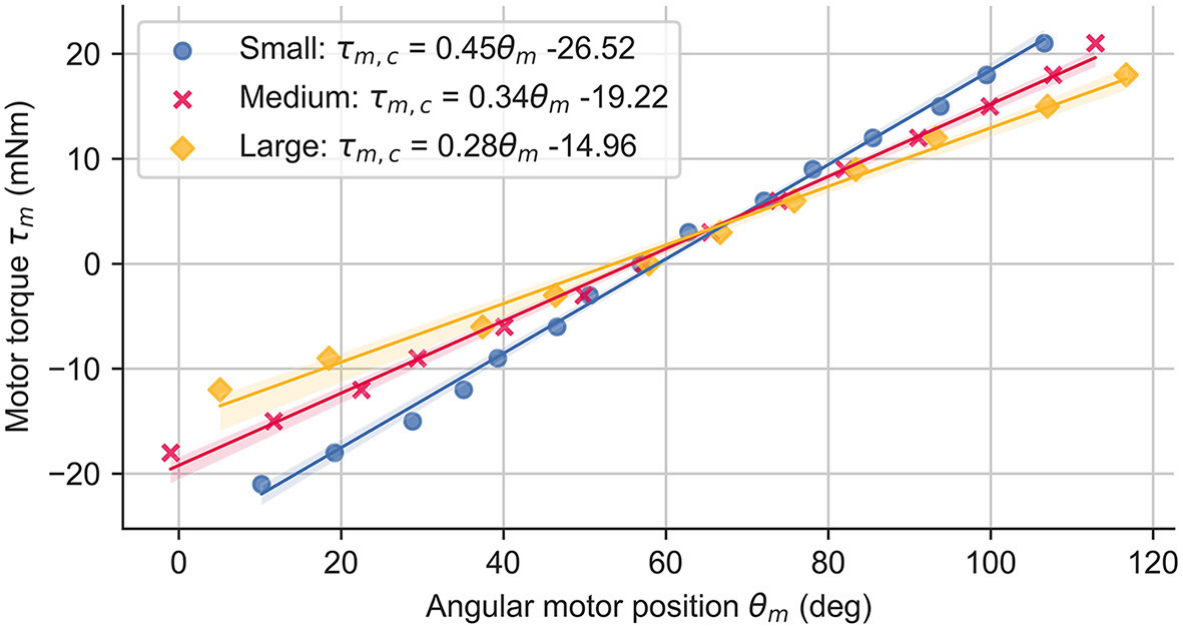
Inherent shell restitution force measurements and resulting compensation torque τ_*m, c*_ computed through linear regression for all three shell sizes.

We increased the device transparency by compensating the inherent restitution force of the printed shell to allow the fingers to move freely when not in contact with virtual objects. For each shell size, we identified the inherent spring constant and offset by applying a constant motor torque in steps of 3 mNm and measuring the resulting angular motor position (and thus the shell deflection). The compensation torque τ_*m, c*_ was then computed as the linear regression of the collected data points (see Figure 2). This leads to a final motor torque in Eq. (6).


(6) $$\begin{array}{l} \tau_{m}=r^{\prime }F+\tau_{m,c}. \end{array}$$

#### 2.1.4 Final prototype

**Figure 3:**
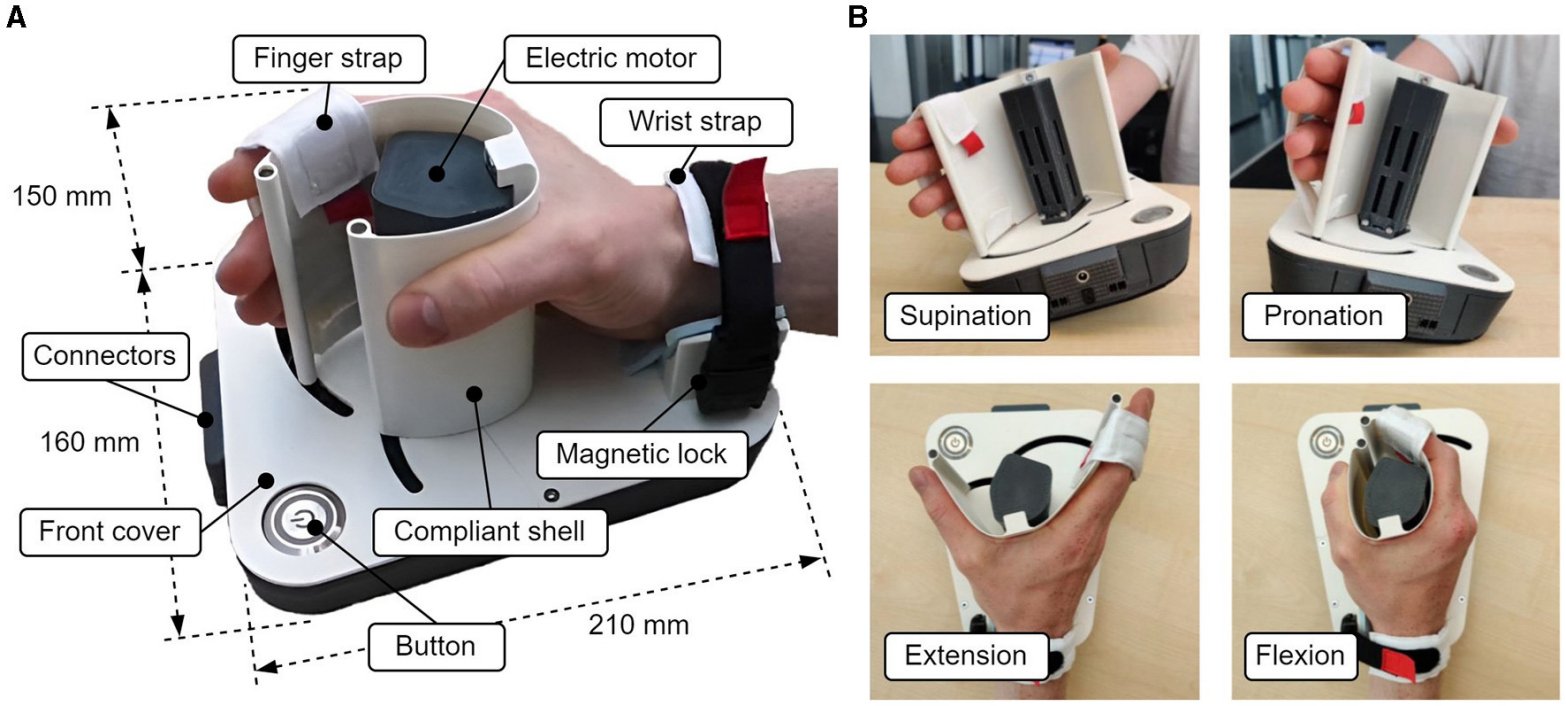
**(A)** Overview of the portable hand trainer. **(B)** Demonstration of pronosupination movements and flexion/extension of the fingers.

The final prototype (Figure 3) consists of a housing in which the transmission and electronic components are placed, a wrist rest, a button with integrated status LED, a DC motor, and the shell. The final prototype has a length of 210 mm, a width of 160 mm, and a height of 150 mm. The range of motion for finger and thumb levers (Figure 1) are approximately 80° and 40°, respectively. The majority of the device was 3D-printed in FDA-approved polylactic acid (PLA) plastic, while a few structural parts were printed in carbon-reinforced PLA or machined out of aluminum or stainless steel (e.g., shafts of the pulleys, vertical support rods at the ends of the shells). This results in a weight of 1030 g without the external power supply and emergency stops. For this study, a right-handed version was manufactured. The cost of one prototype unit without external emergency stop buttons is currently slightly below 1,000 CHF (approx. 1,000 EUR).

Since the endpoints of the three differently sized shells move on different paths, each shell comes with a distinct cover for the frontal upper part of the device, which is fixated on the device with magnets (front cover in Figure 3A). A shell can be exchanged in a few seconds by first pulling it vertically off the device (the vertical support rods can either stay in the shell or be removed separately), then exchanging the front cover and inserting the new shell (see video in Supplementary Material).

The DC motor with an optical encoder (3272CR and IER3 with 4096 pulses, Faulhaber, Germany) is placed vertically outside the main housing and inside the shell, as this allows the most efficient use of space. The resulting maximal continuous force at the fingertips depends on the shell size, 15.7 N (small), 13.6 N (medium), and 11.9 N (large). The device is powered by an external medical-grade 12 V power supply and can be equipped with two emergency stop buttons in series (one for the user and one for a therapist or experimenter). An ESP-32 microcontroller (Espressif Systems, China) with an Escon Module 50/5 motor driver (Maxon, Switzerland) is used to control the device. The embedded control software was written in C++ and is based on the open-source FreeRTOS™ (Amazon, USA) real-time operating kernel, allowing for appropriate scheduling and prioritization of tasks. One of the two cores of the microprocessor runs a dedicated thread for the haptic rendering and motor control at 1 kHz. Games or rehabilitation exercises are executed on a host computer, with which the device communicates through a USB serial connection.

To enable pronosupination movements (i.e., tilting of the device, Figure 3B), the edges of the bottom of the device were rounded. This allows smooth tilting of the device up to 15° to each side, however, the device can easily be tilted more while leaning on its edge. Because of the remaining flat part in the center, the device is still stable when positioned on a flat surface. The tilting angle is measured with an LSM6DSOX (Adafruit, USA) inertial measurement unit (IMU). A wrist strap was added to fix the user's hand in position and to facilitate pronosupination movements. The size of the wrist strap is adjustable via hook and loop and can be opened with a magnetic lock (Fidlock GmbH, Germany). Another strap was attached onto the distal position on the shell to fixate the fingertips (Figure 3A). This strap can be opened with a hook and loop fixation, however, this is not necessarily required as the fingers can simply be sled in.

Before use, the device needs to go through a short calibration step. Upon a three-second press on the device button, a calibration is performed by slowly closing the shell with a proportional-integral (PI) velocity controller and detecting the sudden increase in controller output when the mechanical limit is hit. This calibration step is required to obtain a mapping from the motor shaft angle—measured by an incremental encoder—to the shell endpoint positions, i.e., the opening of the shell. The shell restitution compensation is executed according to the shell size, which must be provided through the host computer prior to the initialization. After completion of the calibration routine, the status LED turns on.

### 2.2 Serious game

#### 2.2.1 Requirements

We complemented our device with a rehabilitation exercise in the form of a serious game. A preliminary version of the game is described in Van Damme et al. (2022). Amongst other literature (e.g., Burke et al., 2009; Lohse et al., 2013; Li et al., 2021), we oriented ourselves during the development in the results of a survey that we performed among clinical personnel (Rätz et al., 2021b). We specified the following requirements for the game in this study: i) Ensure that the device can effectively showcase its available movements—i.e., power grasp and pronosupination movements. ii) The task in the game should resemble ADL, while still being entertaining. iii) To motivate participants, we aimed to incorporate a challenge-based component, e.g., limited game life, time constraints, and a scoring system. iv) The users shall be encouraged to actively extend their fingers during the exercise. v) The game should contain sub-tasks with different degrees of difficulty. In Rätz et al. (2021b), we found that adjustability of difficulty was desired by clinical personnel. We decided to abstain from adjustable settings in this study to keep the main focus on the evaluation of the device. vi) Provide meaningful, congruent, and diverse haptic feedback during interactions with virtual objects in the game.

#### 2.2.2 Game design

**Figure 4:**
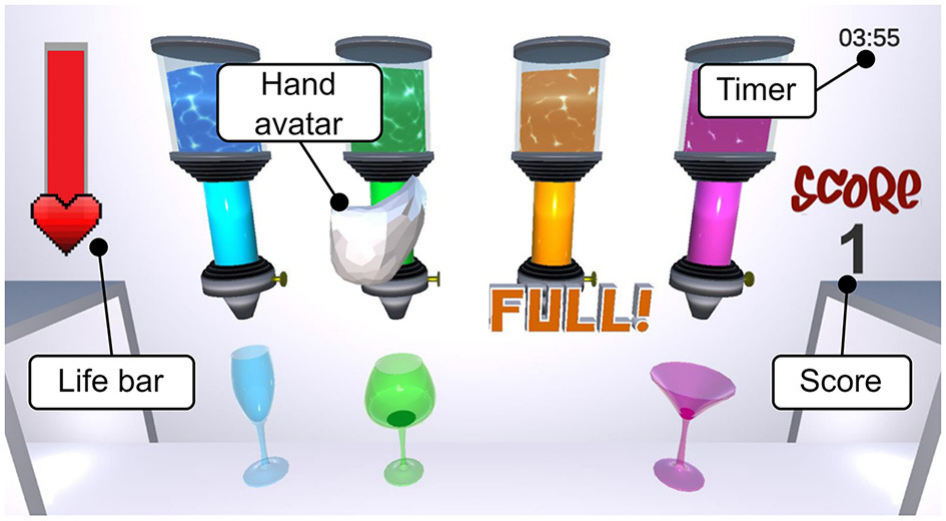
Serious game with four liquid dispensers and hand avatar. The goal is to skillfully squeeze the liquid dispensers to pour liquids into the glasses without spilling any liquid.

A screenshot of the designed serious game that satisfies the aforementioned requirements is shown in Figure 4. The game was developed in Unity3D (Unity Technologies, USA) and represents a cocktail bar with four differently colored liquid dispensers, each one having a glass beneath it. The goal is to fill the glasses by grasping the liquid dispensers with a virtual hand avatar by skillfully squeezing the shell. If the liquid dispensers are grasped too strongly, the liquid starts to spill, which results in lost game life, indicated with a life bar located at the top left of the screen. If the liquid dispensers are not squeezed strongly enough, no or only very little liquid will come out. A timer on the upper right corner of the screen indicates the remaining time. If glasses are overfilled and liquid flows over, the life bar also decreases. For each filled-up glass, the text “Full” appears and a point is added to the user's score, shown on the right of the screen.

The game simulates grasping by mimicking the sensation of physically squeezing a liquid dispenser made of a visco-elastic material. Each liquid dispenser has different characteristics (i.e., stiffness *K* and damping *B* in Eq. 5), reflected in the haptic rendering. When the user squeezes the shell, the virtual hand avatar first moves forward to the liquid dispenser in front of it, and, when further squeezed, the liquid dispenser is grasped. The variable *s*_0_ from Eq. 5 is defined such that the rendering of the virtual wall starts at this point. Importantly, each liquid dispenser possesses different characteristics, i.e., different pairs of stiffness and damping (*K*∈{0.6, 0.1, 0.3, 0.01} N/mm and *B*∈{0.005, 0.001, 0.005, 0} Ns/mm), empirically selected and corresponding to the dispensers from left to right. Notably, the displayed behavior of the liquid matches the haptic rendering, e.g., a liquid dispenser with higher impedance (i.e., higher values of *K* and *B*) contains a sticky, viscous liquid, while a lower impedance indicates a runny liquid.

In the first phase, each one of the four glasses needs to be filled once. To move the virtual hand to grasp different dispensers, the fingers need to be extended, and the device tilted—i.e., performing a pronosupination movement. When the IMU detects tilting of more than 5°, the hand avatar moves one step (i.e., one liquid dispenser) in the corresponding tilting direction. After keeping the device tilted for 0.8 s, the avatar continues moving to the next position and so forth, until the device tilting angle is below 5° again. These values were defined through preliminary testing by the developers. To switch position again after a liquid dispenser has been grasped, the hand must be opened again, necessitating active finger extension as specified in the requirements. Once the first four glasses are filled, glasses start to appear randomly. If the life bar is empty, the score is reset to zero and the first phase starts again.

### 2.3 Usability evaluation

#### 2.3.1 Participants

A total of 13 healthy participants took part in the usability evaluation of our haptic device (six male, six female, and one non-binary; ages between 21 and 64 years; twelve right-handed and one left-handed). Of the 13 participants, three were neurorehabilitation physiotherapists from Rijndam Rehabilitation Center, Rotterdam, the Netherlands. The other ten participants (referred to as non-expert participants in this study) were healthy adults recruited through word of mouth at the Delft University of Technology, Delft, the Netherlands. Following, we refer to participants by pseudonyms T1–T3 (therapists) and N1–N10 (non-expert participants). All participants were naive to the experiment and haptic device. The study was approved by the Human Research Ethics Committee (HREC) of the Delft University of Technology (Application ID: 2216).

#### 2.3.2 Experimental setup and procedure

An unsupervised rehabilitation scenario was reproduced in two different locations. For the non-expert participants, we performed the experiment in a room (approximately 10 m × 5 m) with a table (2.5 m × 1 m) and a height-adjustable chair with backrest. On top of the table, we placed the hand trainer, a laptop, and one emergency stop button. The hand trainer was always placed on the right side beside the laptop, while the emergency stop button was placed on the left side in an easily reachable position. The entire setup was facing a short wall of the room. For the physiotherapists, the experiment was performed at the rehabilitation center in an office (approximately 6 m × 5 m) with a similar setup. A physiotherapist, who was familiar with the device but not involved in its development, led the experiment. One of the device developers was also around to provide support in case of technical difficulties.

The experiment started with obtaining the participants' written consent. Their hand size was then measured and a shell size—i.e., small, medium, large—was suggested by the experimenter according to a predefined size correspondence table. However, participants could switch to a different size after trying the recommended size if desired. The swapping of the shell was performed by the experimenter and is not part of the usability evaluation because in a real-life setting, this would be performed by the therapist and the patient would receive a device where the correct shell is already installed. Five participants felt the most comfortable with the small shell, while the other eight chose the medium size.

After selecting the shell size, participants were equipped with eye-tracking glasses (Tobii Pro Glasses 2, Tobii, Sweden). They were allowed to wear the eye-tracking glasses on top of their prescription glasses. The eye-tracking glasses were calibrated for each user following the manufacturer's guidelines for optimal performance. Participants whose calibration could not be performed successfully due to their prescription glasses were removed from the analysis. The glasses recorded a video of the participant's point of view and a sequence of gaze points (i.e., where they were looking). In addition to the eye-tracking glasses, the experiment was recorded with a video camera, allowing us to measure setup times and identify practical and technical issues after the experiment.

The participants were then invited to sit on the chair and follow the instructions on the laptop screen. They were asked to seek the help of the experimenters only in case of emergency or if they could not continue by themselves. In the case of the non-expert participants, the experimenters moved behind a movable wall equipped with a second emergency stop button but did not leave the room to ensure the participants' safety. At the rehabilitation center, the experimenters positioned themselves diagonally behind the physical therapists to stay out of their line of sight and simulate the minimally supervised scenario while still ensuring the participants' safety with the second emergency stop.

Participants were then asked to follow the instructions presented on the laptop screen through a series of slides related to the device setup, play of the game, and device doffing. The slides related to the device setup instructions included how to turn on the device, how to don the hand, the game instructions, and how to use the emergency button. The device could be turned on by pressing the device button (Figure 3) for at least three seconds. The participants could move to the next instruction slide with a short press of the same device button. After the instructions related to the device setup, a new slide prompted participants to play the game for five minutes. The remaining gaming time was displayed in the upper right corner during the game. When the time was up, a new slide with instructions on turning off the device and releasing the hand appeared. The entire set of instruction slides can be found in the Supplementary material. After the experiment, participants were asked to complete several questionnaires (see Section 2.3.3) and invited to share their experiences in a semi-structured interview. The audio of the interview was recorded for later analysis.

#### 2.3.3 Outcome measures

We defined a variety of quantitative and qualitative outcome measures to assess the usability of the device as well as the participants' motivation and workload. First, the lead experimenter manually recorded the set-up time, i.e., the time required to turn on the device, donning, and doffing. We also noted the number of issues that occurred during the experiment. Hereby we categorized between *practical issues* (e.g., when the participant visibly misunderstood the instructions or did not know how to proceed) and *technical issues* (e.g., issues related to the device or the game). In each case, we further noted whether intervention from the experimenters was required to continue the experiment. In cases where the experimenter did not have a clear view of the participant, the recorded video was consulted *ad hoc*.

We assessed the participants' subjective perception of the system's usability with two questionnaires. We selected the Post-Study Usability Questionnaire (PSSUQ) (Lewis, 2002) for the entire system (i.e., game and device). It consists of 16 seven-point Likert-style items and is divided into three subscales: System Usefulness (i.e., satisfaction, simplicity, and comfort), Information Quality (i.e., if and how relevant information is presented), and Interface Quality (i.e., interaction with the device and game). For an isolated assessment of the device, we additionally employed the shorter System Usability Scale (SUS) questionnaire (Brooke, 1996), which consists of ten five-point Likert-style items. We chose the PSSUQ for the entire system as it exhibits finer granularity and the SUS for the isolated assessment of the device since the PSSUQ contains questions that only make sense in the presence of a software or information component.

The fact that the cognitive capabilities of stroke patients are often affected (e.g., see Mercier et al., 2001) motivated us to also investigate the mental load of our participants when using the system. We utilized the raw NASA Task Load Index (RTLX) (Hart, 2006), a widely used questionnaire in usability testing (Meyer et al., 2021). The RTLX assesses six individual domains, namely the mental, physical, and temporal (i.e., perceived time pressure) demand, the perceived performance, effort (i.e., the effort needed to achieve the performance), and the level of frustration. Each domain is assessed through a single 21-point Likert-style item, whereby zero reflects “very low” (or “perfect” in the performance item) and 20 “very high” (or “failure” in the performance item).

Since motivation is known to be a strong driver of effort and participation in robotic training of stroke patients (Sivan et al., 2014), we also included items from the Interest/Enjoyment and the Perceived Competence subscales of the Intrinsic Motivation Inventory (IMI) (McAuley et al., 1989). All questionnaire scores were normalized to a range from 0 to 100 for a more straightforward interpretation of the results. The PSSUQ and IMI subscale scores for each participant were computed by taking the arithmetic average of the corresponding items, and the overall scores of the PSSUQ and SUS were averaged for all items.

**Figure 5:**
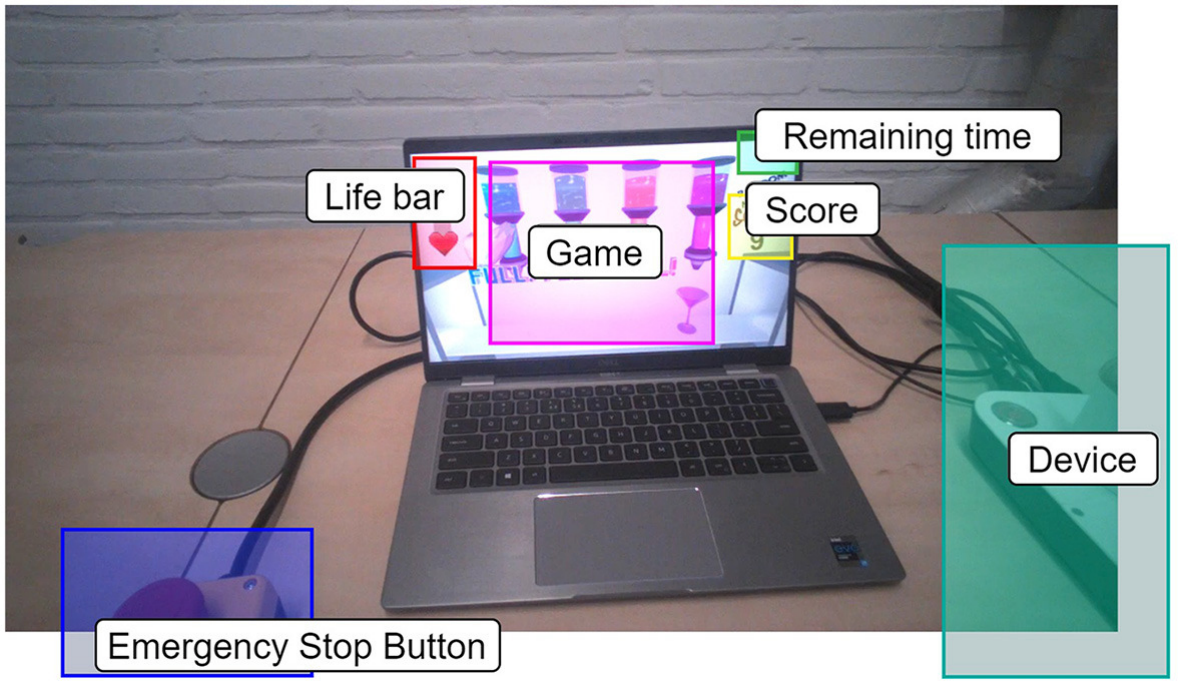
Exemplary frame from the video recorded by the Tobii glasses for participant N8. The six different rectangular areas of interest (AOIs) are highlighted in different colors.

We employed the recorded eye-tracking data, i.e., the participants' points of view and accompanying gaze points, to identify the time participants spent looking at different elements of the experimental setup while playing the game. In the context of usability, the proportion of time spent looking at an element (*gaze point rate*) may reflect the importance of that element or could indicate difficulties in understanding an element (Jacob and Karn, 2003). This was achieved by counting the number of gaze points per participant landing on six different rectangular areas of interest (AOIs, Figure 5), representing elements of the experimental setup: the device, emergency stop, game (i.e., dispensers and glasses), life bar, score, and remaining time. The number of gaze points landing on the different AOIs was determined per participant from the eye-tracking videos using the AOI tool of the Tobii Pro Lab software (version 1.217, Tobii, Sweden). The AOIs were manually adjusted for keyframes, i.e., individual frames of the videos, at the beginning and end of head movements to ensure that the AOIs were accurately placed on top of their corresponding element. The AOIs' positions and sizes were then linearly interpolated between keyframes. We normalized the number of gaze points per participant and AOI *n*_*AOI*_ over the total number of gaze points per participant *n*_*total*_ to remove the effect of unequal dataset sizes between participants (${\hat{n}}_{AOI}=n_{AOI}/n_{total}$). The gaze point rates ${\hat{n}}_{AOI}$ were multiplied by the time spent playing the game (300 s) to calculate the total time participants looked at each of the AOIs.

Finally, we gathered qualitative data through open-ended questions (see Supplementary material) in semi-structured interviews. These questions served as initial prompts to guide the discussion, though the experimenters were free to ask follow-up questions, allowing them to explore topics that seemed particularly important to the individual participant. The audio recordings of the semi-structured interviews were transcribed locally on a computer with a custom software pipeline written in Python. First, a diarization (i.e., partitioning of the audio into segments according to the speaker) was performed with *simple-diarizer* (Simple Diarizer, 2023) using the *xvec* model and spectral clustering. The verbatim transcription was then performed based on *faster-whisper* (Faster Whisper, 2023), which is a re-implementation of the automatic speech recognition *Whisper* (Radford et al., 2023). We employed the pretrained *medium* size model. Afterwards, the transcriptions were manually checked and corrected analogous to the audio recordings. A thematic analysis was then performed to determine the principal themes (i.e., recurring patterns, opinions, and ideas) that emerged from the interviews. This methodology involves a systematic examination of the data, wherein text segments are designated descriptive labels known as codes. These codes with the accompanying text segments are then categorized into cohesive themes, which are subsequently summarized and reported. For a comprehensive description of the procedure, please refer to Braun and Clarke (2008).

## 3 Results

All 13 participants except participant N10 completed all steps of the experiment. The experiment with this participant was ended prematurely by the experimenters when the participant was playing the game due to technical problems with the device (see Table 1); however, participant N10 completed the rest of the experiment (i.e., questionnaires and interview) according to the protocol.

**Table 1:** Technical and practical issues during setup and game play.

**Type**	**Issue**	**Occurences**	**Help**
Practical	Participant started the setup before reading the instructions.	1	
	Participant did not open the magnetic fastener of the wrist strap during setup.	5	
	Participant grasped the shell multiple times roughly, risking device damage.	1	
	The button was pressed too lightly and did not activate.	1	
	Participant did not follow the instructions and asked how to continue.	1	x
	Participant asked about the life bar of the game.	1	x
	Participant asked about the pronosupination movement.	1	x
Technical	The game did not react properly to the participant's movements.	1	
	Device button did not react to the participant input.	1	x
	Calibration failed; restart was required.	1	x
	Encoder calibration lost: Undesired shell movements (experiment stop, N10).	1	x

Help indicates whether assistance from the experimenters was required.

### 3.1 Setup time, technical issues and practical issues

**Figure 6:**
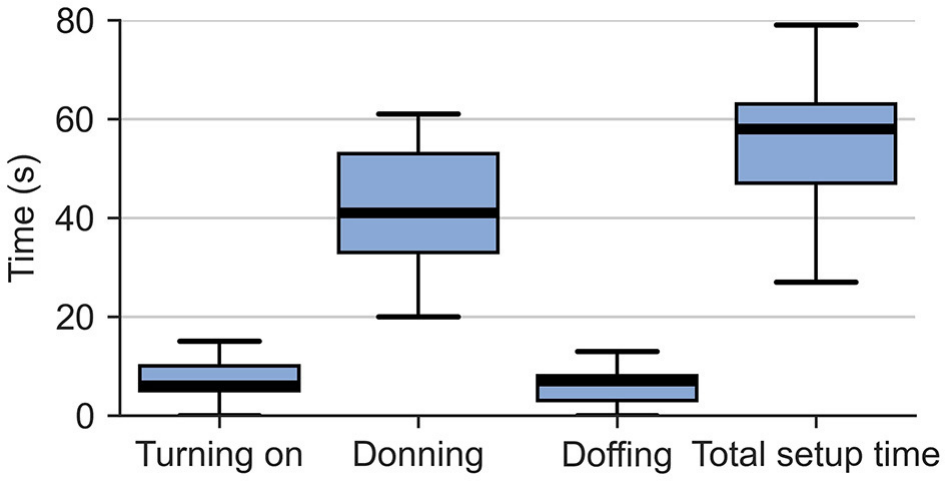
Box plots of the setup time, subdivided into turning on, donning, and doffing. The whiskers extend to ±1.5 inter-quartile range (IQR) from the nearest hinge.

The setup time measurements are depicted in Figure 6. The overall median time (first quartile, third quartile) that participants spent with the device setup was 58 (47, 63) s. In particular, turning on the device took 6 (5, 10) s, while the subsequent donning took 41 (33, 53) s. Finally, the doffing was again relatively quick, with a duration of 7 (3, 8) s.

The encountered technical and practical issues are summarized in Table 1. Overall, ten practical and four technical issues were observed. With five occurrences, the most observed issue was participants not properly using the magnetic wrist strap. One technical error (N10) led to the ending of the experiment for safety reasons since the technical root cause for this event was unknown at that time.

### 3.2 Questionnaires

**Figure 7:**
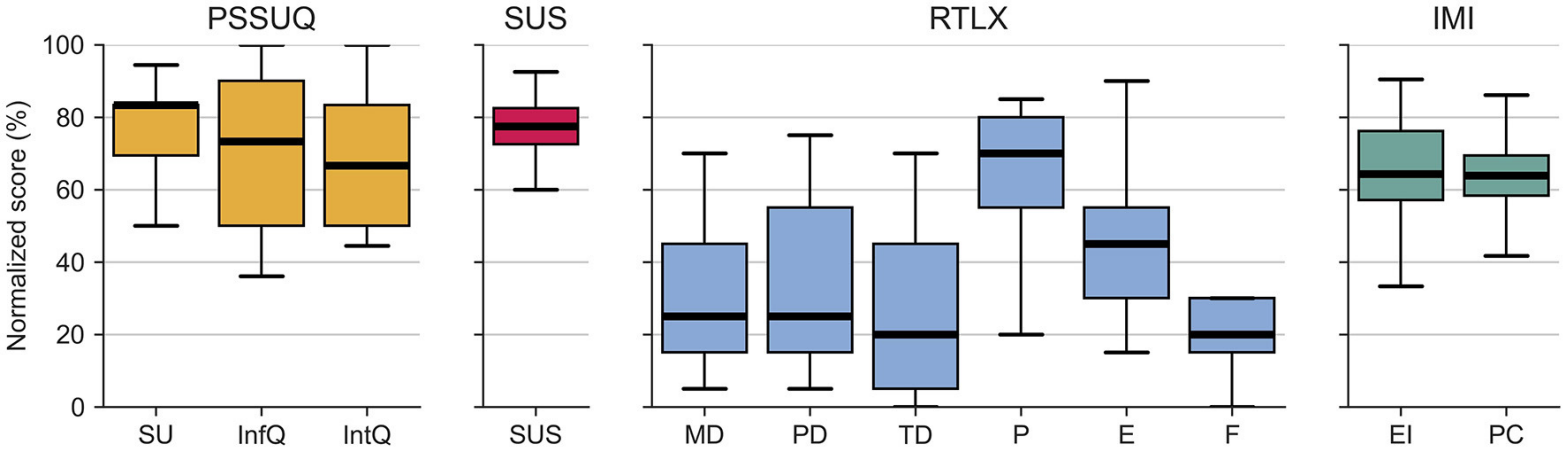
Normalized scores from the questionnaires. **PSSUQ:** SU, System Usefulness; InfQ, Information Quality; IntQ, Interface Quality, **SUS:** SUS, Total score; **RTLX:** MD, Mental Demand; PD, Physical Demand; TD, Temporal Demand; P, Performance; E, Effort; F, Frustration; **IMI:** EI, Enjoyment/Interest; PC, Perceived Competence. The whiskers extend to 1.5 IQR from the nearest hinge.

The normalized scores of the questionnaires are summarized in Figure 7. Because of the ordinal nature of the results from the various questionnaires (Sullivan and Artino, 2013), we represent the central tendency using the median with first and third quartiles.

Regarding the usability questionnaires, the PSSUQ questionnaire, which was applied to the entire system, achieved an overall rating of 70.2 (65.6, 85.6) out of 100. Hereby, the System Usefulness subscale scored the highest with 83.3 (69.4, 83.3), followed by the Information Quality with 73.3 (50.0, 90.0) and Interface Quality with 66.7 (50.0, 83.3). The isolated device usability rating from the SUS achieved a score of 77.5 (72.5, 82.5). SUS values of 50.9–71.4, 71.4–85.5, and 85.5–90.9 correspond to OK–good, good–excellent, and excellent–best-imaginable usability, respectively according to Bangor et al. (2009). Note that previous studies have shown that the PSSUQ and the SUS questionnaires are highly correlated (Vlachogianni and Tselios, 2023).

The assessment with the RTLX showed a mental demand of 25.0 (15.0, 45.0), a physical demand of 25.0 (15.0, 55.0), and a temporal demand of 20.0 (5.0, 45.0). Furthermore, it revealed that participants rated their performance with 70.0 (55.0, 80.0), which they achieved with a perceived effort of 45.0 (30.0, 55.0). Hereby, they rated their frustration level as 20.0 (15.0, 30.0) out of 100. In general, low values of the RTLX items indicate a low workload, except for the performance item, where a high value indicates good perceived performance.

Finally, regarding motivation, the overall IMI Interest/Enjoyment subscale score reached 64.3 (57.1, 76.2) out of 100 and the Perceived Competence subscale reached a score of 63.9 (58.3, 69.4). High scores in the IMI subscales relate to high enjoyment and high perceived competence, respectively.

### 3.3 Gaze point rates per AOI

**Figure 8:**
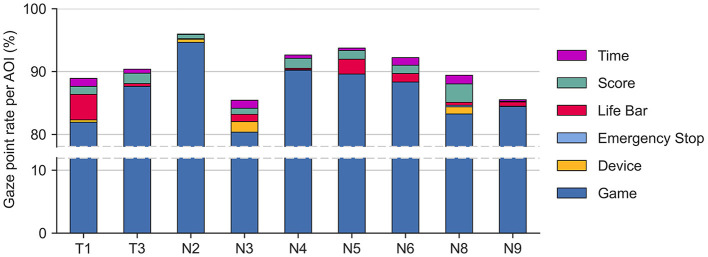
Gaze point rates per AOI for each participant with eye-tracking (nine out of the 13).

The results of the gaze point rate per AOI are shown in Figure 8. Two of the eye-tracking datasets were removed due to failed calibration procedures (N1, T2), one caused by technical issues with the data (faulty battery, N7), and one because of the premature termination of playing the game (N10), leaving nine out of 13 datasets. The screen area with the cocktail glasses and dispensers (i.e., the game AOI) obtained the highest normalized hit rate with 87.0% (4.3%) (average and standard deviation). Notably, participants T1 and N5 spent a considerable amount of time looking at the life bar (12.2 s and 7.1 s), while participants N3 and N8 spent more time looking at the device when compared to their peers (5.0 s and 3.5 s, respectively). Overall, the hit rates of 0.42% (0.57%) on the device and 0.03% (0.07%) on the emergency stop with resulting average duration of only 1.27 s and 0.097 s, respectively, were low in comparison with other AOIs.

### 3.4 Semi-structured interviews

The thematic analysis led to the classification of 495 quotations, resulting in the assignment of 86 codes, which we then organized into seven groups: *General Impressions, Pronosupination Movements, Instructions, Game, Comfort, Grasping with Haptic Rendering*, and *Application & Clinical Use*. Following, we present the main findings for each group with examples of supporting participant statements.

#### 3.4.1 General impressions

The participants liked the sleek and simplistic design of the device. The majority of the participants appreciated having only one button for all functions, as it simplified the user experience and reduced the need to remember multiple buttons. One participant expressed concerns about accidentally turning the device off.

“*It's quite portable, it's looking sleek, it has nice curves”* (T1)

“*I think it's very simple so that's great.”* (N6)

“*I like that there's only one button, because it's just easy”* (N4)

The weight and size of the device was generally considered acceptable. Some suggested making it slightly lighter, while others thought it provided stability.

“*I think it's nice that it's heavy when you have to move it, because then you really feel that it's rolling through.”* (N4)

#### 3.4.2 Pronosupination movements

Seven participants mentioned that the device tilting action to move between dispensers felt clunky and less responsive than expected. They struggled with the step-by-step movement of the hand avatar when tilting and were unsure if the hand needed to stay tilted to move multiple dispenser positions.

“*And the turning to the left and right was very... It was taking steps. I thought it was more fluid, but it was taking steps.”* (T2)

Furthermore, some participants stated that the tilting felt counter-intuitive at first as the design itself did not look as it was supposed to be tilted.

“*It didn't feel very intuitive when I was moving it left and right. Because I would imagine if it's a device that's supposed to rotate it would have something at the bottom that's not flat.”* (N6)

#### 3.4.3 Instructions

The reported feelings about the setup and game instructions were mixed. While some participants complimented the simplicity, seven participants mentioned that the instructions were not clear enough and raised concerns about the cognitive load, especially for users with potential cognitive impairments. They recommended simplifying the instructions, making them less information-dense. Furthermore, it was repeatedly suggested that step-by-step video demonstrations or looping animations might be more informative and easier to follow.

“*Very clear. And concise. Yeah no it was clear.”* (N5)

“*I think it's more understandable if I see a 5-minute video and see this is the procedure, then there is no need to read something.”* (N8)

In particular, for the magnetic wrist strap, the participants wished to obtain more detailed information about the exact opening mechanism. Several participants were initially confused about the magnetic mechanism of the wrist strap. Some did not realize that it could be opened and instead released the adjacent hook and loop. It was also mentioned that the color coding of the parts could be improved (e.g., finger strap), and should be chosen more carefully to represent their respective importance during the setup. For example, the wrist strap locking mechanism should be visually more highlighted than the finger strap adjustment as it is required to be opened every time during setup, while the finger strap only needs to be adjusted occasionally.

“*The only problem I had was with the wrist strap. It says open the lock which I interpreted as just open the hook and loop.”* (N8)

“*Yes, but there's a red strap here so at first I was just like this because I read quickly and I didn't really understand [...] maybe this [finger strap red part] shouldn't be highlighted more than this [wrist fixation].”* (N5)

Participant T2 mentioned having read only a little bit of the instructions, and Participant N8 admitted clicking through the instructions, without following them.

#### 3.4.4 Game

The game was generally perceived as fun and enjoyable to play for the given time. Although some participants struggled to some extent with the pronosupination movements to move the virtual hand sideways, the game appeared to be intuitive for most participants.

“*The game, yes, it was funny. I wouldn't play it for hours, of course, but I think it's intuitive and fun.”* (N5)

Five participants reported that the concept of the life bar was not fully understood or that the life bar was not even noticed for the majority of the time. It was suggested to make the life bar visually more dominant or to explain it better during the instructions.

“*The position of the bar needs to be closer to what's happening. Or there needs to be some visual connection.”* (N6)

Yet, few participants noted that the game was boring or could quickly become boring. In this context, some participants expressed their disappointment that the score was not saved and that there was no high score they could beat. It was suggested that a more competitive setting—even if it is just beating one's own score—would increase their motivation and interest in the game. Furthermore, more levels with increased difficulty would help to maintain motivation during longer sessions. The timer was mostly appreciated as a motivational element, although one participant perceived it as stressful.

“*It also wasn't really clear to me what my previous score was, so what score should I beat? Because it was a fun game to play, I would like to be competitive.”* (N1)

“*Just shortly doing it is okay but playing it longer will be very boring for me.”* (T2)

#### 3.4.5 Comfort

Participants found the device generally comfortable and safe. Nevertheless, concerns about the wrist position and angle during prolonged use were raised, especially for persons with a paretic upper limb. Due to the height of the device, the hand was in an elevated position with respect to the elbow, resulting in a slight ulnar abduction.

“*It is quite comfortable. I was like in a relaxing pose. It was not stressing my hand, it is also very smooth and it is not too tight.”* (N2)

“*The position of my wrist was a little bit uncomfortable, I think because it was elevated from the table.”* (N8)

Two participants found the finger strap adjustment slightly finicky due to the limited space to attach the hook and loop on the shell. One participant desired to have the finger strap in a more proximal position. One participant pointed out that the thumb's position was somewhat unclear, and three suggested that a thumb strap might be helpful during extension movements.

#### 3.4.6 Grasping with haptic rendering

Participants generally found the grasping motion easy to perform. Most of them appreciated the realistic grasping sensation and how the haptic feedback correlated with their actions.

“*At the beginning I was looking at the device to see where my fingers were, but at some point, I was just not looking anymore because of the haptic feedback. It was nice.”* (N5)

“*Really cool, how the grasping really works nicely with the feedback, it really felt like I had some nice feedback, yeah it worked well”* (N1)

However, a few also reported that the visuals played a predominant role in their interaction and expressed the need for more prominent and informative haptic feedback.

“*I don't know how much I would have been able to tell the difference without the visual aid because I don't know if like my brain was so sensitive to what's happening with my hand. I think those visuals were super important.”* (N6)

“*I did not feel that a lot. I saw a lot with the drops, but I did not feel very different things.”* (T3)

#### 3.4.7 Application and clinical use

All participants stated that they would feel comfortable using the device themselves in an unsupervised environment in the hypothetical scenario of undergoing upper-limb rehabilitation. Two out of the three participating therapists noted that they would use it with their patients, while one was not sure yet. The therapists saw potential applications either in early rehabilitation, group therapy, or home rehabilitation—in particular for patients with reduced tactile or proprioceptive sensibility.

“*I think when they have sensibility problems it's very difficult to give the right force to hold a glass or something. So people do that or it's too loose and it falls. So I think with this device you can maybe learn a little bit more and normally we do that with grabbing things. So I think it can be useful for that kind of problems.”* (T3)

One therapist noted that stroke patients might benefit from adjustable assistance during the exercise. One mentioned that an initial assessment of patients' range of motion and available grasping force could be used to adjust the device and the game. Moreover, therapists highlighted the importance of variation during the rehabilitation training and suggested increasing the number of available exercises/games.

## 4 Discussion

### 4.1 We evolved our concept into a safe, aesthetic, and functional prototype

We developed a minimally-actuated device to meet the need for cost-effective haptic upper-limb training devices for minimally supervised or unsupervised neurorehabilitation. We realized a device that is inherently safe, suitable for a variety of hand sizes, and that can provide meaningful haptic feedback during the grasping of virtual objects by combining a compliant shell design with highly back-drivable actuation. We refined the device's appearance, and also added a passive DoF for wrist pronosupination, a movement highly recommended by therapists (Rätz et al., 2021b), by allowing the entire device to be tilted around its longitudinal axis. The combination of passive and active degrees of freedom is in line with the recommendations of Forbrigger et al. (2023a), who suggested this concept to reduce cost while still providing high functionality. We thus satisfied all the required device improvements that we defined based on the first concept (see Section 2.1.1).

Our novel hand trainer is complemented by a serious game that challenges users to fill virtual cocktail glasses using simulated liquid dispensers with different haptic behaviors, highlighting the haptic capabilities of our device. Thereby, the difficulty of successfully filling the glass without spilling any liquid depends on the simulated liquid and varies across the different dispensers. The task mimics a scenario akin to ADL, as it requires precise grasping, force dosing and timing to succeed. Moreover, the game promotes finger extension, as users must open their hand before switching between liquid dispensers using pronosupination movements.

When compared to the state of the art—represented by similar devices like the PoRi (Wolf et al., 2022) or the ReHandyBot (Articares Pte Ltd, Singapore)—our innovation exhibits a distinct advantageous combination of portability, intrinsic safety, and setup simplicity. Functional differences are that the PoRi is more lightweight and can be freely moved in space by patients with advanced proximal upper-limb functions, while our device sits stably on a surface, making it also accessible for more impaired patients. The ReHandyBot, already available on the market, offers actuated pronosupination, although at the cost of increased complexity. While other studies consider devices of more than 50 kg still portable (e.g., Sivan et al., 2014), we agree with Lu et al. (2011) that a portable device should be compact and lightweight enough to be easily transported to patients' homes—preferably by patients themselves—and low-cost. The affordability of our device is enabled by the combination of one active with one passive DoF and a readily available low-cost microcontroller and IMU. Moreover, most parts could be manufactured from technical plastics as we demonstrated by the mostly 3D-printed prototype. Currently, the main cost-driving elements are the high-end electric motor and motor driver, as they make up for more than 50% of the device's price.

To evaluate our design, we performed a usability study in a simulated unsupervised environment with 13 healthy participants, of whom three were physiotherapists from Rijndam Rehabilitation, Rotterdam, the Netherlands. This experience allowed us to gain valuable insights and information to note what needs to be dropped, added, kept, and improved in the following design iteration.

### 4.2 Lessons learned from the usability evaluation

#### 4.2.1 Our device requires less than one minute to set up

The overall median set-up time—including turning on, donning, and doffing—remained below one minute. This is five times lower than the maximum setup time of robotic devices that therapists are willing to spend in inpatient rehabilitation (Rätz et al., 2021b). While the requirements in terms of setup time for home rehabilitation remain to be investigated, if we assume that they are of similar magnitude as those in a clinical setting, we feel confident that our device setup time is acceptable for home rehabilitation users. The very short doffing times observed once the participants understood how the straps work, already indicate that it is likely that our device could be donned and doffed even faster with more experience. Yet, it remains to be evaluated how stroke survivors—especially those suffering from spasticity and not being able to extend their fingers—will be able to accomplish the device setup.

#### 4.2.2 Overall, our haptic device is perceived as highly usable and intuitive

The entire system, i.e., taking into account the device and game, achieved an overall median PSSUQ rating of 70.2 out of 100, indicating good usability, while the isolated device usability rating from the SUS achieved a score of 77.5, considered to correspond to good—excellent usability based on the ranges defined in Bangor et al. (2009). These values are in line with those from other studies of devices for similar applications. For example, the HandyBot was attributed a SUS score of 76.3 and 85.0 for the device itself and the GUI respectively (Ranzani et al., 2023). The user interface of the ReHapticKnob was rated 85.0 and two accompanying haptic games with 76.3 and 68.8 (Ranzani et al., 2021). The MERLIN device scored 71.9 in a home rehabilitation feasibility study (Guillén-Climent et al., 2021). Lastly, a SUS score of 77.5 was reported for the GripAble device in a usability study with Parkinson's disease patients (Saric et al., 2022).

The semi-structured interviews allowed us to gain a deep insight into participants' opinions. In general, the device was considered user-friendly and participants highlighted that the device looked sleek, portable and simple, thereby endorsing the overall concept. Interestingly, participants almost did not mention the shell during the interviews, suggesting that the interaction appeared to be natural and intuitive. This is supported by the results from the eye-tracking data, which show that participants did not look much at the device itself while playing the game. It seems that it was not necessary to often look at the device after donning it, indicating a generally intuitive and seamless human-device interaction. Importantly, the gaze rate on the emergency stop was marginal, possibly reflecting that participants felt safe during playing or indicating a high level of involvement in the game.

The results from the RTLX questionnaire, which reflect the participant's perceived workload during the experiment, seem to endorse the idea that the system was perceived as intuitive. With a median score of 20 and no data point higher than 30, the frustration level of the participants appears acceptable given that they used the device the first time. Furthermore, the median score of the mental, physical, and time demands were lower than 25, although with a larger dispersion. Yet, while lower values of the RTLX are preferable (except the inverted Performance item) for rehabilitation device interfaces (Ranzani et al., 2021), it can not be generally stated that mental, physical, and temporal demand, as well as effort, should be as low as possible for games or exercises. On the contrary, for example, to achieve a high exercise intensity, a larger (perceived) effort is typically desirable (Eston et al., 1987; Church et al., 2021). To promote neuroplasticity—which is the ultimate goal of this device—the performance should be high enough to keep the user motivated, but low enough to provide room for improvement (Guadagnoli and Lee, 2004). The perceived median performance score of 70 in combination with the perceived effort score of 45 indicates that the difficulty might have been appropriate for the skill level of the healthy participants. This is supported by the perceived competence subscale of the IMI, which is in line with the RTLX perceived performance item.

#### 4.2.3 We should invest in game personalization

While some participants reported that they loved the game, some found it very boring. This seems to be reflected in the resulting score of the Enjoyment/Interest subscale of the IMI (64.3 over 100), which indicates a good but not high median intrinsic motivation of the participants during the experiment (Reynolds, 2007). As a comparison, the MERLIN device scored 85.7 in a home rehabilitation setting over a duration of a few weeks (Guillén-Climent et al., 2021). We presume that our study's lower score might be explained by the varying interests of the participants in the game, potentially influencing their intrinsic motivation. The diversity of participants' feelings and opinions not only highlights the need to improve our game further but also shows that multiple, different games would be a necessity for an at-home study with patients. A collection of interesting and diverse games is a prerequisite for successful home rehabilitation. Indeed, it has been observed that the usage times of robotic devices at home are low when the patients reported a lack of complexity and enjoyment in the games (Sivan et al., 2014). In particular for rehabilitation with stroke patients, it will be important to provide difficulty levels that are tailored to each patient's abilities (Colombo et al., 2007).

Moreover, multiple participants pointed out that a more elaborate scoring system (e.g., personal high score) could increase their motivation. Both the interviews and eye-tracking showed varying utilization and understanding of the life bar (that reflects performance), for which we see two reasons: i) Although the life bar was indicated in the instruction slides, we did not explicitly mention how it works. The time of five minutes might have been too short for some participants to implicitly learn the relation between life bar and spilling. ii) The interviews revealed that some participants did not notice the life bar.

#### 4.2.4 There is room for improvement in the wrist fixation and the passive pronosupination degree of freedom

Twelve out of the thirteen participants were able to perform the setup of the device and play the game with no or minimal intervention. Yet, we identified a few practical and technical issues. With regard to practical issues, i.e., those related to misconception or incorrect manipulation, we found that five of the eleven occurrences stemmed from participants having difficulties with the magnetic wrist lock. While this specific practical issue did not require the intervention of the experimenters, it points to a usability issue. This was not expected, as this part was indeed designed to facilitate the setup. This issue is supported by the comments gathered from the semi-structured interviews that pointed out that the instructions regarding the wrist fixation might have not been clear enough.

The pronosupination passive DoF also gathered the attention of participants. We did not only find that it caused one of the practical issues, but multiple participants reported that the movements were not straightforward. One reason could be that the rounding of the bottom edges of the device is uniform along its length—i.e., cylindrical with the center flat part. This is in contrast to literature that describes pronosupination movements as rolling movements of a cone, with its center being the elbow (Kapandji, 1982). Thus, the rolling of the device might not correspond to natural, physiological pronosupination. Another reason could be that the flat bottom that we designed for stability seemed actually to have discouraged users from tilting the device. The pronosupination issue might have been further aggravated by the wrist position, which could become uncomfortable for prolonged use, according to the interviews. Indeed, the wrist is in a slight ulnar abducted position due to the elevated hand position with respect to the elbow.

#### 4.2.5 The haptic rendering is generally well perceived

Participants generally appreciated the realistic haptic sensation and how the haptic feedback correlated with their grasping actions. Yet, a few participants mentioned that they did not consciously notice or use the haptic feedback. For this, we suggest four possible explanations: i) The haptic forces were not strong enough. ii) The haptic feedback worked well and was very coherent with the game. Therefore, participants did not actually notice that the haptic rendering forces were generated artificially. iii) Participants might have confounded the expected inherent springiness of the shell with the haptic rendering. iv) Participants subconsciously noticed the haptic feedback but did not perceive it as informative as they might have relied on the visual feedback as for example suggested by the answers of N1 and N6.

Although it is likely that some participants have mistaken the haptic rendering for the inherent compliance of the shell, points (i), (ii) and (iv) would require further investigation to be confirmed or disproved, for example in a within-subject study with haptic and non-haptic conditions. We can, however, comment on point (i): It is indeed possible that stiffness and damping values might have been chosen too low. A stiffness of 2 N/mm is required for an object to be perceived as stiff (Massie and Salisbury, 1994), while our stiffest object was only 0.6 N/mm. The chosen values were thought to well represent the deformable dispensers. However, it might have been beneficial to choose higher values or at least to accentuate the impact when touching a dispenser (e.g., with more distinct values of the *K* and *B* gains or vibratory cues).

#### 4.2.6 Instructions are of critical importance for devices in minimally supervised environments

The other practical issues only occurred once and included instances where participants either did not adhere to the provided instructions or manipulated the device too early/late. We also noted confusion related to the game, in particular to the life bar and the pronosupination movements. This could indicate that parts of the instructions might not have been clear. This is supported by several statements from the semi-structured interviews. Indeed, we believe that unclear instructions were the main reason behind some of the low scores in the Information Quality subscale of the PSSUQ results. The score dispersion of the Information Quality is the highest among the PSSUQ subscales, showing that participants' perceptions of this aspect were very diverse, i.e., some were completely satisfied with the provided information, while others desired improvements in the provided information.

This brings us to an important learning for device development for unsupervised settings: The instructions are equally important as the device and the exercise themselves. In hindsight, we must acknowledge that we focused on the device and game during the development. This calls for the need to include other stakeholders in all design phases, such as cognitive psychologists.

#### 4.2.7 The device could benefit from improvements to make it more robust

Although technical issues may be unfortunate at first sight—for example, for participant N10, who was not able to play the games for the full five minutes—they are an inherent aspect of early testing and a valuable opportunity for improving the device. The particular incident with N10 was most likely caused by slippage of the large gear pulley (pulley with diameter *d*_2_ in Figure 1) on its axle due to insufficient clamping. This caused a misalignment of the motor encoder. The other technical issues necessitate further reliability testing of the software and the implementation of online error-checking routines and appropriate measures. For example, a failed calibration can easily be detected by driving the shell along its entire range of motion and comparing the resulting distance with the expected distance.

### 4.3 Study limitations

Our study has some limitations and shortcomings. First, we did not include stroke patients in this first usability study. While the inclusion of stroke patients in usability evaluations is undeniably important, the involvement of non-expert participants and therapists can also contribute indispensable insights in the early stages of device development. Following the double diamond design process model, after the first phases of *discover* and *define*, the iterative phases of *design* and *deliver* start, where new designs are created and evaluated by end users (Design Council, 2005). Ideally, the patients should be included in all these phases. Yet, the bureaucratic work required to involve patients in testing is long and tedious, requiring approvals from the local ethics committees every time a modification/improvement is made, which slows down the design process. Therefore, intermediate evaluation steps with healthy non-expert participants and therapists serving as proxies allow already assessing basic functionality, general user experience, and initial usability challenges that might not be exclusive to stroke patients. This helps to detect usability problems early, thus allowing faster convergence to more appropriate solutions, saving time, and reducing the burden on patients.

Second, an inherent drawback of our experimental design is that the usability of the device itself might have been confounded with the quality of the instructions. It has been shown that there is a positive significant correlation between the quality of user instructions and perceived product quality (Gök et al., 2019). Therefore, unclear instructions might have aggravated the perception of usability issues. However, in the case of this study, our set of rather minimalist instructions might actually have helped to extract the maximum amount of information from the experiment.

Third, the findings of our study could be limited by the participants' awareness of the experimenters' presence as the unsupervised scenario was only simulated. While this setup allowed intervention for practical or technical issues, it could have affected the participants' behavior when compared to a fully unsupervised setting.

### 4.4 Next steps in our human-centered design approach

In this first usability study, we gathered valuable information, recommendations, and points for improvements to be exploited in the next design iteration. In short, we plan to work on: i) Adapt the bottom of the device and the wrist fixation to facilitate the pronosupination movements while guaranteeing physiological positioning of the wrist. ii) Develop more games with different difficulty levels, and include an improved scoring system (e.g., personal high score). iii) Accentuate the haptic rendering to provide better noticeable variations between different game objects. This might include the implementation of more advanced techniques to further promote sensorimotor learning, such as haptic error modulation (Marchal-Crespo et al., 2019; Basalp et al., 2021). iv) Change the modality of instructions: Instead of slides, we will explore the use of video instructions. Moreover, we might perform checks to see if the user performed the correct action before continuing to the subsequent one. v) Further increase the portability of our system by removing the emergency stop buttons, potentially replacing the external power supply with a battery, and switching to wireless communication. This step will also necessitate making the device more robust and reliable. vi) Integrate an absolute encoder and automatic detection of the installed shell size to avoid the currently necessary calibration sequence. vii) Implement an assessment routine that allows to determine the user's range of motion and grasping force. viii) Further lower the cost of the device, for example by replacing the motor and motor driver with a lower-cost solution or the redesign of complicated components. On this note, the general robustness might also be further improved in prospect of potential future large-scale studies.

Gathering patient feedback—potentially also in a longitudinal study—will be our main focus after realizing the aforementioned improvements. The combination of group therapy with home rehabilitation (where patients use the exact same device) has been suggested as a promising way of efficiently increasing therapy dosage (McCabe et al., 2019) and could present a suitable use case for the next round of usability testing.

With respect to the possible commercialization of the device, the distribution and support will become key factors that need to be considered. Moreover, we will investigate various financial models to ensure the economic viability of such a relatively low-cost device once it is ready for commercialization. It has been shown that innovations with potentially high societal impact but lower economic value—e.g., medical low-cost devices such as the one presented in this study—are notoriously difficult to obtain investments (Allers et al., 2023). Thus, we must ensure that our device is not only low-cost but, first of all, cost-efficient, i.e., it must not only hold its therapeutic premise but also provide an economic benefit to the health care system and investors.

### 4.5 Conclusion

We presented the second iteration of a novel minimally-actuated haptic hand trainer for minimally supervised and unsupervised rehabilitation of patients with acquired brain injury, as well as an accompanying serious game. The introduction of a novel compliant shell mechanism allowed us to design a device that is simple and provides intuitive and intrinsically safe physical human-device interaction.

Following a human-centered iterative development approach, we performed a thorough analysis of the prototype's usability with therapists and healthy non-expert users. In a simulated unsupervised scenario, we asked the participants to set up the device and play a game based on a set of written instructions. Our mixed-method approach allowed us to gain insights into usability issues of our prototype. While the testing showed good overall usability of the device and the game, we identified various areas of improvement, such as the wrist fixation, the pronosupination movements, and instructions.

Our prototype shows promise for use in both minimally supervised therapy and unsupervised home rehabilitation. We are looking forward to further improving our device to deploy it with neurological patients and contribute to the democratization of robotic rehabilitation in order to improve the quality of life of especially vulnerable patients.

## Data availability statement

The original contributions presented in the study are included in the article/Supplementary material, further inquiries can be directed to the corresponding author.

## Ethics statement

The studies involving humans were approved by Human Research Ethics Committee (HREC) of the Delft University of Technology (Application ID: 2216). The studies were conducted in accordance with the local legislation and institutional requirements. The participants provided their written informed consent to participate in this study.

## Author contributions

RR: Conceptualization, Data curation, Formal analysis, Investigation, Methodology, Project administration, Software, Visualization, Writing – original draft, Writing – review & editing. AR: Conceptualization, Data curation, Formal analysis, Investigation, Methodology, Supervision, Visualization, Writing – original draft, Writing – review & editing. NC-G: Conceptualization, Data curation, Formal analysis, Investigation, Writing – review & editing. GR: Conceptualization, Methodology, Project administration, Resources, Supervision, Validation, Writing – review & editing. LM-C: Conceptualization, Funding acquisition, Methodology, Project administration, Resources, Supervision, Validation, Writing – original draft, Writing – review & editing.

## References

[B1] Akbari A. Haghverd F. Behbahani S. (2021). Robotic home-based rehabilitation systems design: from a literature review to a conceptual framework for community-based remote therapy during COVID-19 pandemic. Front. Robot. AI 8, 1–34. 10.3389/frobt.2021.612331 34239898 PMC8258116

[B2] Allers S. Eijkenaar F. van Raaij E. M. Schut F. T. (2023). The long and winding road towards payment for healthcare innovation with high societal value but limited commercial value: A comparative case study of devices and health information technologies. Technol. Soc. 75, 102405. 10.1016/j.techsoc.2023.102405

[B3] Bangor A. Kortum P. Miller J. (2009). Determining what individual SUS scores mean: Adding an adjective rating scale. J. Usabil. Stud. 4, 114–123. 10.5555/2835587.2835589

[B4] Basalp E. Wolf P. Marchal-Crespo L. (2021). Haptic training: Which types facilitate (re)learning of which motor task and for whom Answers by a review. IEEE Trans. Haptics 1412, 3104518. 10.1109/TOH.2021.3104518 34388095

[B5] Biddiss E. A. Chau T. T. (2007). Upper limb prosthesis use and abandonment. Prosthetics & *Orthot. Int*. 31, 236–257. 10.1080/03093640600994581 17979010

[B6] Bolognini N. Russo C. Edwards D. J. (2016). The sensory side of post-stroke motor rehabilitation. Restor. Neurol. Neurosci. 34, 571–586. 10.3233/RNN-150606 27080070 PMC5605470

[B7] Braun V. Clarke V. (2008). Using thematic analysis in psychology, qualitative research in psychology. J. Chem. Inf. Model. 3, 77–101. 10.1191/1478088706qp063oa

[B8] Brooke J. (1996). SUS: “A ‘quick and dirty' usability scale,” in *Usability Evaluation in Industry, Number* (Boca Raton: CRC Press), 207–212.

[B9] Bullock I. M. Zheng J. Z. Rosa S. D. L. Guertler C. Dollar A. M. (2013). Grasp frequency and usage in daily household and machine shop tasks. IEEE Trans. Haptics 6, 296–308. 10.1109/TOH.2013.6 24808326

[B10] Burke J. W. McNeill M. D. Charles D. K. Morrow P. J. Crosbie J. H. McDonough S. M. (2009). Optimising engagement for stroke rehabilitation using serious games. Visual Comp. 25, 1085–1099. 10.1007/s00371-009-0387-4

[B11] Camillieri S. (2019). A paradigm shift for acute rehabilitation of stroke. J. Stroke Med. 2, 17–22. 10.1177/2516608519848948

[B12] Chen Y. Abel K. T. Janecek J. T. Chen Y. Zheng K. Cramer S. C. (2019). Home-based technologies for stroke rehabilitation: a systematic review. Int. J. Med. Informat. 123, 11–22. 10.1016/j.ijmedinf.2018.12.001 PMC6814146 30654899

[B13] Chi N. F. Huang Y. C. Chiu H. Y. Chang H. J. Huang H. C. (2020). Systematic review and meta-analysis of home-based rehabilitation on improving physical function among home-dwelling patients with a stroke. Arch. Phys. Med. Rehabil. 101, 359–373. 10.1016/j.apmr.2019.10.181 31689417

[B14] Church G. Smith C. Ali A. Sage K. (2021). What is intensity and how can it benefit exercise intervention in people with stroke? a rapid review. Front. Rehabilitat. Sci. 2, 722668. 10.3389/fresc.2021.722668 PMC9397782 36188814

[B15] Ciortea V. M. Motoac I. Ungur R. A. Borda I. M. Ciubean A. D. Irsay L. (2021). Telerehabilitation a viable option for the recovery of post-stroke patients. Applied Sciences 11:10116. 10.3390/app112110116

[B16] Colombo R. Pisano F. Mazzone A. Delconte C. Micera S. Carrozza M. C.. (2007). Design strategies to improve patient motivation during robot-aided rehabilitation. J. Neuroeng. Rehabil. 4, 3. 10.1186/1743-0003-4-3 17309790 PMC1805445

[B17] Cramer S. C. Dodakian L. Le V. See J. Augsburger R. McKenzie A.. (2019). Efficacy of home-based telerehabilitation vs in-clinic therapy for adults after stroke: a randomized clinical trial. JAMA Neurol. 76, 1079–1087. 10.1001/jamaneurol.2019.1604 31233135 PMC6593624

[B18] Design Council (2005). A Study of the Design Process “*The Double Diamond*”. Available online at: https://www.idi-design.ie/content/files/ElevenLessons_Design_Council_2.pdf

[B19] Eston R. G. Davies B. L. Williams J. G. (1987). Use of perceived effort ratings to control exercise intensity in young healthy adults. Eur. J. Appl. Physiol. Occup. Physiol. 56, 222–224. 10.1007/BF00640648 3569229

[B20] Faster Whisper (2023). faster-whisper. Available online at: https://github.com/guillaumekln/faster-whisper (accessed November 11, 2023).

[B21] Feigin V. L. Brainin M. Norrving B. Martins S. Sacco R. L. Hacke W.. (2022). World Stroke Organization (WSO): global stroke fact sheet 2022. Int. J. Stroke 17, 18–29. 10.1177/17474930211065917 34986727

[B22] Fong J. Crocher V. Tan Y. Oetomo D. Mareels I. (2017). “EMU: A transparent 3D robotic manipulandum for upper-limb rehabilitation,” in 2017 International Conference on Rehabilitation Robotics (ICORR) (London: IEEE), 771–776. 10.1109/ICORR.2017.8009341 28813913

[B23] Forbrigger S. DePaul V. G. Davies T. C. Morin E. Hashtrudi-Zaad K. (2023a). Home-based upper limb stroke rehabilitation mechatronics: challenges and opportunities. Biomed. Eng. Online 22, 67. 10.1186/s12938-023-01133-8 37424017 PMC10329803

[B24] Forbrigger S. Liblong M. Davies T. DePaul V. Morin E. Hashtrudi-Zaad K. (2023b). Considerations for at-home upper-limb rehabilitation technology following stroke: Perspectives of stroke survivors and therapists. J. Rehabilitat. Assist. Technol. Eng. 10, 205566832311718. 10.1177/20556683231171840 37124709 PMC10134106

[B25] Garrett J. W. (1971). The adult human hand: some anthropometric and biomechanical considerations. Human Factors. 13, 117–131. 10.1177/001872087101300204 5550584

[B26] Gassert R. Dietz V. (2018). Rehabilitation robots for the treatment of sensorimotor deficits: a neurophysiological perspective. J. Neuroeng. Rehabil. 15, 46. 10.1186/s12984-018-0383-x 29866106 PMC5987585

[B27] Gök O. Ersoy P. Börühan G. (2019). The effect of user manual quality on customer satisfaction: the mediating effect of perceived product quality. J. Product & *Brand Manageme*. 28, 475–488. 10.1108/JPBM-10-2018-2054

[B28] Goldberg J. H. Wichansky A. M. (2003). “Eye tracking in usability evaluation,” in The Mind's Eye (London: Elsevier), 493–516.

[B29] Guadagnoli M. A. Lee T. D. (2004). Challenge point: a framework for conceptualizing the effects of various practice conditions in motor learning. J. Mot. Behav. 36, 212–224. 10.3200/JMBR.36.2.212-224 15130871

[B30] Guillén-Climent S. Garzo A. Mu noz-Alcaraz M. N. Casado-Adam P. Arcas-Ruiz-Ruano J. Mejías-Ruiz M.. (2021). A usability study in patients with stroke using MERLIN, a robotic system based on serious games for upper limb rehabilitation in the home setting. J. Neuroeng. Rehabil. 18, 1–16. 10.1186/s12984-021-00837-z 33622344 PMC7901008

[B31] Haakenstad A. Irvine C. M. S. Knight M. Bintz C. Aravkin A. Y. Zheng P.. (2022). Measuring the availability of human resources for health and its relationship to universal health coverage for 204 countries and territories from 1990 to 2019: a systematic analysis for the Global Burden of Disease Study 2019. Lancet 399, 2129–2154. 10.1016/S0140-6736(22)00532-3 35617980 PMC9168805

[B32] Hackett M. L. Vandal A. C. Anderson C. S. Rubenach S. E. (2002). Long-term outcome in stroke patients and caregivers following accelerated hospital discharge and home-based rehabilitation. Stroke 33, 643–645. 10.1161/str.33.2.643 11823686

[B33] Handelzalts S. Ballardini G. Avraham C. Pagano M. Casadio M. Nisky I. (2021). Integrating tactile feedback technologies into home-based telerehabilitation: opportunities and challenges in light of COVID-19 pandemic. Front. Neurorobot. 15, 617636. 10.3389/fnbot.2021.617636 33679364 PMC7925397

[B34] Hart S. G. (2006). NASA-task load index (NASA-TLX); 20 years later. Proc. Human Factors Ergon. Soc. 2006, 904–908. 10.1177/154193120605000909

[B35] Hesse S. Heß A. Werner C.C Kabbert N. Buschfort R. (2014). Effect on arm function and cost of robot-assisted group therapy in subacute patients with stroke and a moderately to severely affected arm: a randomized controlled trial. Clin. Rehabil. 28, 637–647. 10.1177/0269215513516967 24452706

[B36] Hyakutake K. Morishita T. Saita K. Fukuda H. Shiota E. Higaki Y.. (2019). Effects of home-based robotic therapy involving the single-joint hybrid assistive limb robotic suit in the chronic phase of stroke: a pilot study. Biomed Res. Int. 2019, 5462694. 10.1155/2019/5462694 31011576 PMC6442446

[B37] Jacob R. J. Karn K. S. (2003). “Eye tracking in human-computer interaction and usability research,” in The Mind's Eye (London: Elsevier).

[B38] Kapandji I. A. (1982). Physiology of the Joints, Volume 1, Upper Limb 5th edition.

[B39] Kwakkel G. Kollen B. J. Van der Grond J. V. Prevo A. J. (2003). Probability of regaining dexterity in the flaccid upper limb: impact of severity of paresis and time since onset in acute stroke. Stroke 34, 2181–2186. 10.1161/01.STR.0000087172.16305.CD 12907818

[B40] Kwakkel G. van Peppen R. Wagenaar R. C. Wood Dauphinee S. Richards C. Ashburn A.. (2004). Effects of augmented exercise therapy time after stroke. Stroke 35, 2529–2539. 10.1161/01.STR.0000143153.76460.7d 15472114

[B41] Lai S.-M. Studenski S. Duncan P. W. Perera S. (2002). Persisting consequences of stroke measured by the stroke impact scale. Stroke 33, 1840–1844. 10.1161/01.STR.0000019289.15440.F2 12105363

[B42] Lambercy O. Lehner R. Chua K. Wee S. K. Rajeswaran D. K. Kuah C. W. K.. (2021). Neurorehabilitation from a distance: can intelligent technology support decentralized access to quality therapy? Front. Robot. AI 8, 1–9. 10.3389/frobt.2021.612415 34026855 PMC8132098

[B43] Lewis J. R. (2002). Psychometric evaluation of the PSSUQ using data from five years of usability studies. Int. J. Hum. Comput. Interact. 14, 463–488. 10.1207/S15327590IJHC143&amp;4_11

[B44] Li L. Fu Q. Tyson S. Preston N. Weightman A. (2021). A scoping review of design requirements for a home-based upper limb rehabilitation robot for stroke. Top. Stroke Rehabil. 00, 1–15. 10.1109/ICIEA51954.2021.9516098 34281494

[B45] Lohse K. Shirzad N. Verster A. Hodges N. Van Der Loos H. F. (2013). Video games and rehabilitation: Using design principles to enhance engagement in physical therapy. J. Neurol. Phys. Ther. 37, 166–175. 10.1097/NPT.0000000000000017 24232363

[B46] Lu E. C. Wang R. H. Hebert D. Boger J. Galea M. P. Mihailidis A. (2011). The development of an upper limb stroke rehabilitation robot: identification of clinical practices and design requirements through a survey of therapists. Disab. Rehabilitat. Assist. Technol. 6, 420–431. 10.3109/17483107.2010.544370 21184626

[B47] Maramba I. Chatterjee A. Newman C. (2019). Methods of usability testing in the development of eHealth applications: a scoping review. Int. J. Med. Inform. 126, 95–104. 10.1016/j.ijmedinf.2019.03.018 31029270

[B48] Marchal-Crespo L. Tsangaridis P. Obwegeser D. Maggioni S. Riener R. (2019). Haptic error modulation outperforms visual error amplification when learning a modified gait pattern. Front. Neurosci. 13, 61. 10.3389/fnins.2019.00061 30837824 PMC6390202

[B49] Massie T. H. Salisbury J. K. (1994). “The PHANTOM haptic interface: a device for probing virtual objects threee enabling observations three necessary criteria for an effective interface,” in ASME Winter Annual Meeting, Symposium on Haptic Interfaces for Virtual Environment and Teleoperator Systems. Dynamic Systems and Control: Volume 55, no. 1 (American Society of Mechanical Engineers).

[B50] McAuley E. D. Duncan T. Tammen V. V. (1989). Psychometric properties of the intrinsic motivation inventoiy in a competitive sport setting: a confirmatory factor analysis. Res. Q. Exerc. Sport 60, 48–58. 10.1080/02701367.1989.10607413 2489825

[B51] McCabe J. P. Henniger D. Perkins J. Skelly M. Tatsuoka C. Pundik S. (2019). Feasibility and clinical experience of implementing a myoelectric upper limb orthosis in the rehabilitation of chronic stroke patients: a clinical case series report. PLoS ONE 14, 1–12. 10.1371/journal.pone.0215311 30978249 PMC6461279

[B52] Mercier L. Audet T. Hebert R. Rochette A. Dubois M.-F. (2001). Impact of motor, cognitive, and perceptual disorders on ability to perform activities of daily living after stroke. Stroke 32, 2602–2608. 10.1161/hs1101.098154 11692024

[B53] Metzger J.-C. Lambercy O. Chapuis D. Gassert R. (2011). “Design and characterization of the ReHapticKnob, a robot for assessment and therapy of hand function,” in 2011 IEEE/RSJ International Conference on Intelligent Robots and Systems (San Francisco: IEEE), 3074–3080.

[B54] Meyer J. T. Gassert R. Lambercy O. (2021). An analysis of usability evaluation practices and contexts of use in wearable robotics. J. Neuroeng. Rehabil. 18, 1–16. 10.1186/s12984-021-00963-8 34886902 PMC8656061

[B55] Meyer J. T. Tanczak N. Kanzler C. M. Pelletier C. Gassert R. Lambercy O. (2023). Design and validation of a novel online platform to support the usability evaluation of wearable robotic devices. Wearable Technol. 4, 31. 10.1017/wtc.2022.31 38487781 PMC10936320

[B56] NICE (2023). “Stroke rehabilitation in adults (update),” in Number October. London: National Institute for Health and Care Excellence. 38635800

[B57] Pandian S. Arya K. N. Davidson E. W. (2012). Comparison of Brunnstrom movement therapy and motor relearning program in rehabilitation of post-stroke hemiparetic hand: A randomized trial. J. Bodyw. Mov. Ther. 16, 330–337. 10.1016/j.jbmt.2011.11.002 22703742

[B58] Pettypiece C. E. Goodale M. A. Culham J. C. (2010). Integration of haptic and visual size cues in perception and action revealed through cross-modal conflict. Exp. Brain Res. 201, 863–873. 10.1007/s00221-009-2101-1 19949777

[B59] Radford A. Kim J. W. Xu T. Brockman G. McLeavey C. Sutskever I. (2023). “Robust speech recognition via large-scale weak supervision,” in International Conference on Machine Learning (PMLR), 28492–28518.

[B60] Ranzani R. Albrecht M. Haarman C. J. Koh E. Devittori G. Held J. P.. (2023). Design, characterization and preliminary usability testing of a portable robot for unsupervised therapy of hand function. Front. Mechan. Eng. 8, 1–17. 10.3389/fmech.2022.1075795

[B61] Ranzani R. Eicher L. Viggiano F. Engelbrecht B. Held J. P. O. Lambercy O.. (2021). Towards a platform for robot-assisted minimally-supervised therapy of hand function: design and pilot usability evaluation. Front. Bioeng. Biotechnol. 9, 2021.01.12.21249685. 10.3389/fbioe.2021.652380 33937218 PMC8082072

[B62] Rätz R. Conti F. Müri R. M. Marchal-Crespo L. (2021a). A novel clinical-driven design for robotic hand rehabilitation: combining sensory training, effortless setup, and large range of motion in a palmar device. Front. Neurorobot. 15, 1–22. 10.3389/fnbot.2021.748196 34987371 PMC8721892

[B63] Rätz R. Müri R. M. Marchal-Crespo L. (2021b). “Assessment of clinical requirements for a novel robotic device forupper-limb sensorimotor rehabilitation after stroke,” in Proceedings of the 5th International Conference on Neurorehabilitation (ICNR2020), eds. D. Torricelli, M. Akay, and J. L. Pons (Vigo: Springer International Publishing).

[B64] Rätz R. Van Damme N. Marchal-Crespo L. Aksöz E. A. (2023). WO2023222678A1 Sensomotoric Hand Therapy Device (Patent), 5th Edn. Churchill Livingstone.

[B65] Renner C. I. Outermans J. Ludwig R. Brendel C. Kwakkel G. Hummelsheim H. (2016). Group therapy task training versus individual task training during inpatient stroke rehabilitation: A randomised controlled trial. Clin. Rehabil. 30, 637–648. 10.1177/0269215515600206 26316552

[B66] Reynolds L. (2007). “Measuring intrinsic motivations,” in Handbook of Research on Electronic Surveys and Measurements, R. A. Reynolds, R. Woods, and J. D. Baker (Pennsylvania: IGI Global), 170–173.

[B67] Rozevink S. G. van der Sluis C. K. Hijmans J. M. (2021). HoMEcare aRm rehabiLItatioN (MERLIN): preliminary evidence of long term effects of telerehabilitation using an unactuated training device on upper limb function after stroke. J. Neuroeng. Rehabil. 18, 141. 10.1186/s12984-021-00934-z 34538246 PMC8449998

[B68] Saric L. Knobel S. E. Pastore-Wapp M. Nef T. Mast F. W. Vanbellingen T. (2022). Usability of two new interactive game sensor-based hand training devices in Parkinson's disease. Sensors 22, 16. 10.3390/s22166278 36016039 PMC9416263

[B69] Schaarup C. Hartvigsen G. Larsen L. B. Tan Z. H. Arsand E. Hejlesen O. K. (2015). Assessing the potential use of eye-tracking triangulation for evaluating the usability of an online diabetes exercise system. Stud. Health Technol. Inform. 216, 84–88. 26262015

[B70] Schneider E. J. Lannin N. A. Ada L. Schmidt J. (2016). Increasing the amount of usual rehabilitation improves activity after stroke: a systematic review. J. Physiother. 62, 182–187. 10.1016/j.jphys.2016.08.006 27637769

[B71] Scott S. H. (2004). Optimal feedback control and the neural basis of volitional motor control. Nat. Rev. Neurosci. 5, 532–544. 10.1038/nrn1427 15208695

[B72] Simple Diarizer (2023). simple_diarizer. Available online at: https://github.com/cvqluu/simple_diarizerr (accessed November 11, 2023).

[B73] Sivan M. Gallagher J. Makower S. Keeling D. Bhakta B. O'Connor R. J.. (2014). Home-based Computer Assisted Arm Rehabilitation (hCAAR) robotic device for upper limb exercise after stroke: Results of a feasibility study in home setting. J. Neuroeng. Rehabil. 11, 1. 10.1186/1743-0003-11-163 25495889 PMC4280043

[B74] Sugawara A. T. Ramos V. D. Alfieri F. M. Battistella L. R. (2018). Abandonment of assistive products: assessing abandonment levels and factors that impact on it. Disabil. Rehabilitat. Assist. Technol. 13, 716–723. 10.1080/17483107.2018.1425748 29334475

[B75] Sullivan G. M. Artino A. R. (2013). Analyzing and interpreting data from likert-type scales. J. Grad. Med. Educ. 5, 541–542. 10.4300/JGME-5-4-18 24454995 PMC3886444

[B76] Swanson V. A. Johnson C. Zondervan D. K. Bayus N. McCoy P. Ng Y. F. J.. (2023). Optimized home rehabilitation technology reduces upper extremity impairment compared to a conventional home exercise program: a randomized, controlled, single-blind trial in subacute stroke. Neurorehabilitat. Neural Repair. 15, 4596832211469. 10.1177/15459683221146995 36636751 PMC9896541

[B77] Tollár J. Nagy F. Csutorás B. Prontvai N. Nagy Z. Török K.. (2021). High frequency and intensity rehabilitation in 641 subacute ischemic stroke patients. Arch. Phys. Med. Rehabil. 102, 9–18. 10.1016/j.apmr.2020.07.012 32861668

[B78] Tollár J. Vetrovsky T. Széphelyi K. Csutorás B. Prontvai N. ács P.. (2023). Effects of 2-year-long maintenance training and detraining on 558 subacute ischemic stroke patients' clinical motor symptoms. Med. Sci. Sports & *Exerc*. 55, 607–613. 10.1249/MSS.0000000000003092 36730024

[B79] Van Damme N. Ratz R. Marchal-Crespo L. (2022). “Towards unsupervised rehabilitation: development of a portable compliant device for sensorimotor hand rehabilitation,” in IEEE International Conference on Rehabilitation Robotics 2022-July:25–29 (Rotterdam: IEEE). 10.1109/ICORR55369.2022.9896556 36176098

[B80] Vlachogianni P. Tselios N. (2023). Perceived Usability Evaluation of Educational Technology Using the Post-Study System Usability Questionnaire (PSSUQ ): *A Systematic Review*.

[B81] Ward N. S. Brander F. Kelly K. (2019). Intensive upper limb neurorehabilitation in chronic stroke: Outcomes from the Queen Square programme. J. Neurol. Neurosurg. Psychiat. 90, 498–506. 10.1136/jnnp-2018-319954 30770457

[B82] Wilson P. H. Rogers J. M. Vogel K. Steenbergen B. McGuckian T. B. Duckworth J. (2021). Home-based (virtual) rehabilitation improves motor and cognitive function for stroke patients: a randomized controlled trial of the Elements (EDNA-22) system. J. Neuroeng. Rehabil. 18, 165. 10.1186/s12984-021-00956-7 34823545 PMC8613521

[B83] Wittmann F. Held J. P. Lambercy O. Starkey M. L. Curt A. Höver R.. (2016). Self-directed arm therapy at home after stroke with a sensor-based virtual reality training system. J. Neuroeng. Rehabil. 13, 1–10. 10.1186/s12984-016-0182-1 27515583 PMC4982313

[B84] Wolf K. Mayr A. Nagiller M. Saltuari L. Harders M. Kim Y. (2022). PoRi device: portable hand assessment and rehabilitation after stroke. Automatisierungstechnik 70, 1003–1017. 10.1515/auto-2022-0037

[B85] Wolf S. L. Biology C. Sahu K. Bay R. C. Buchanan S. Healthcare S.. (2015). The HAAPI (home arm assistance progression initiative) trial: - a novel robotics delivery approach in stroke rehabilitation. Neurorehabil. Neural Repair 29, 958–968. 10.1177/1545968315575612 25782693 PMC4573760

[B86] Zbytniewska-Mégret M. Salzmann C. Kanzler C. M. Hassa T. Gassert R. Lambercy O.. (2023). The evolution of hand proprioceptive and motor impairments in the sub-acute phase after stroke. Neurorehabil. Neural Repair. 37, 823–836. 10.1177/15459683231207355 37953595 PMC10685702

