# Supplementary material for: Designing for usability: development and evaluation of a portable minimally-actuated haptic hand and forearm trainer for unsupervised stroke rehabilitation
Source: Front Neurorobot. 2024 Apr 4;18:1351700. doi: 10.3389/fnbot.2024.1351700 (PMC11024237; doi:10.3389/fnbot.2024.1351700)

Supplementary Material

1 STANDARDIZED QUESTIONAIRES

1.1 System Usability Scale

Table S1. System Usability Scale (SUS) questions.

Nr.	Question
Q1:	I think that I would like to use this system frequently.
Q2:	I found the system unnecessarily complex.
Q3:	I thought the system was easy to use.
Q4:	I think that I would need the support of a technical person to be able to use this system.
Q5:	I found the various functions in this system were well integrated.
Q6:	I thought there was too much inconsistency in this system.
Q7:	I would imagine that most people would learn to use this system very quickly.
Q8:	I found the system very cumbersome to use.
Q9:	I felt very confident using the system.
Q10:	I needed to learn a lot of things before I could get going with this system.

1.2 Intrinsic Motivation Inventory

Table S2. Interest/Enjoyment subscale of the Intrinsic Motivation Inventory (IMI) questionnaire.

Nr.	Question
Q1:	I enjoyed doing this activity very much.
Q2:	This activity was fun to do.
Q3:	I thought this was a boring activity. (R)
Q4:	This activity did not hold my attention at all. (R)
Q5:	I would describe this activity as very interesting.
Q6:	I thought this activity was quite enjoyable.
Q7:	While I was doing this activity, I was thinking about how much I enjoyed it.

Table S3. Perceived competence subscale of the Intrinsic Motivation Inventory (IMI) questionnaire.

Nr.	Question
Q8:	I think I am pretty good at this activity.
Q9:	I think I did pretty well at this activity, compared to other participants.
Q10:	After working at this activity for awhile, I felt pretty competent.
Q11:	I am satisfied with my performance at this task.
Q12:	I was pretty skilled at this activity.
Q13:	This was an activity that I couldn't do very well. (R)

1.3 Post-study System Usability Questionnaire

Table S4. System Usefulness subscale of the Post-study System Usability Questionnaire (PSSUQ

Nr.	Question
Q1:	Overall, I am satisfied with how easy it is to use this system.
Q2:	It was simple to use this system.
Q3:	I was able to complete the tasks and scenarios quickly using this system
Q4:	I felt comfortable using this system.
Q5:	It was easy to learn to use this system.
Q6:	I believe I could become productive quickly using this system.

Table S5. Information Quality subscale of the Post-study System Usability Questionnaire (PSSUQ

Nr.	Question
Q7:	The system gave error messages that clearly told me how to fix problems.
Q8:	Whenever I made a mistake using the system, I could recover easily and quickly.
Q9:	The information provided with this system was clear.
Q10:	It was easy to find the information I needed.
Q11:	The information was effective in helping me complete the tasks and scenarios.
Q12:	The organization of information on the system screens was clear.

Table S6. Interface Quality subscale of the Post-study System Usability Questionnaire (PSSUQ

Nr.	Question
Q13:	The interface of this system was pleasant.
Q14:	I liked using the interface of this system.
Q15:	This system has all the functions and capabilities I expect it to have.
Q16:	Overall, I am satisfied with this system.

1.4 NASA Task Load Index

Table S7. NASA Task Load Index questionnaire.

Nr.	Question
Q1:	How mentally demanding was the task?
Q2:	How physically demanding was the task?
Q3:	How hurried or rushed was the pace of the task?
Q4:	How successful were you in accomplishing what you were asked to do?
Q5:	How hard did you have to work to accomplish your level of performance?
Q6:	How insecure, discouraged, irritated, stressed, and annoyed were you?

2 SEMI-STRUCTURED INTERVIEWS

Table S8. Set of initial questions for the semi-structured interviews. The questions marked with (T) only applied to therapists. Note that the order and occurrence of the questions was adjusted according to the answers of the participant.

Nr.	Question
Q1:	What do you think of the design of the prototype device?
Q2:	What do you think of the serious game?
Q3:	Do you think the instructions are clear enough?
Q4:	Would you use it unsupervised?
Q5:	Would you change anything about the design of the prototype device or the game?
Q6:	Do you think this device could be useful for patients? What kind of patients/pathologies/symptoms? (T)
Q7:	Do you think the use of this device could improve patient performance at activities of daily living? How? (T)
Q8:	Do you think the instructions are easy-understandable for a patient (with possible cognitive impairment)? (T)
Q9:	Do you think it might be interesting to consider and/or reduce the weight of the device?
Q10:	Would you add any other movement to the device taking into account the patient's common needs? (T)
Q11:	What would be missing from this device for you to use it in your clinic with your patients? (assuming that the quantity of cables and emergency buttons are reduced). What features would you like? (T)

3 INSTRUCTIONS

1 Turn on the device

- Do not touch the shell. Do not put the hand in the device.
- Press the device button for at least 3 seconds and release to turn it on. The LED indicator will be blinking.
- The device will now automatically perform a calibration and a self-check. The shell will move slowly during the calibration.

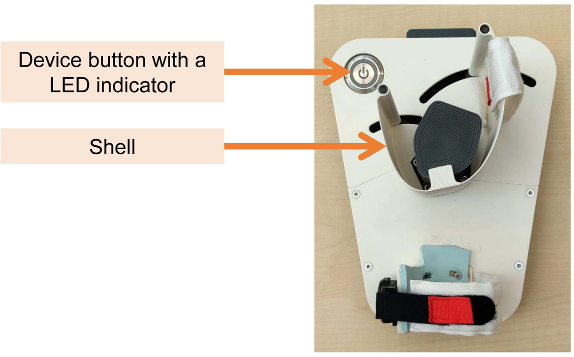

To see the next step, push the device button once quickly. ➡

2 Setup hand

- Pull back the locking mechanism of the wrist strap to open it.
- Place your right hand around the outside of the shell. You can adjust the finger strap if needed.
- Close the wrist strap and adjust if necessary. The wrist strap locking mechanism is magnetic, so you will hear a click sound when it locks.

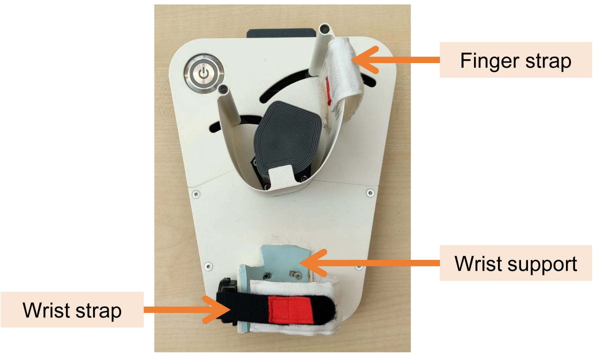

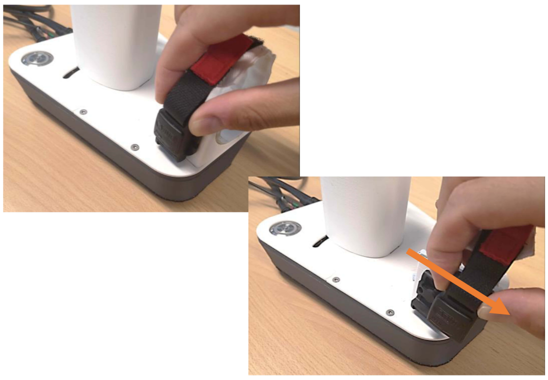

To see the next step, push the device button once quickly. ➡

3 Game instructions

➢ Squeeze all the bottles to fill the glasses trying not to spill the liquid.

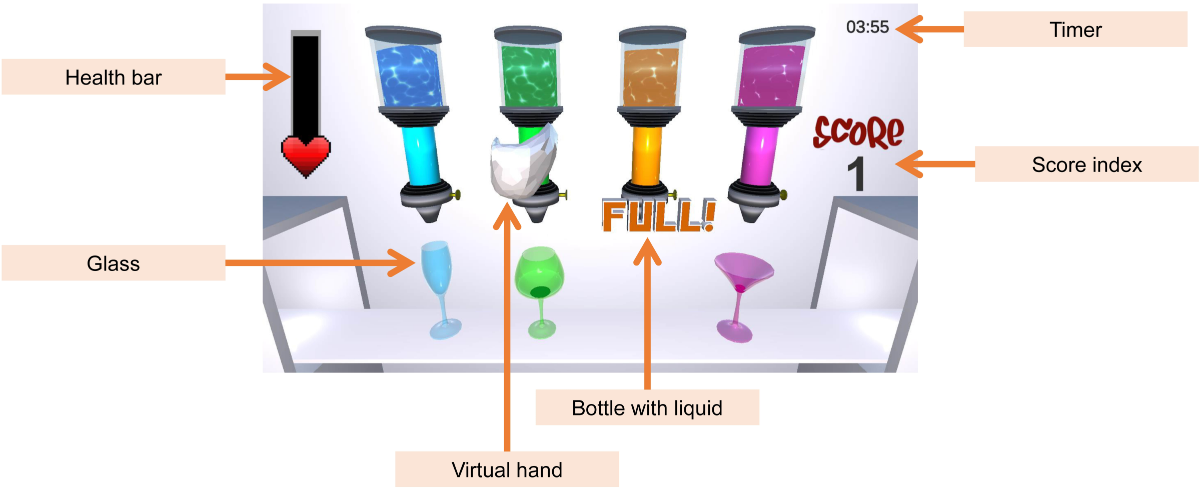

To see the next step, push the device button once quickly.

3 Game instructions

➢ Grasp the shell to squeeze the bottles. Each bottle will feel different. Adjust your grasp force to control the flow of the liquid.

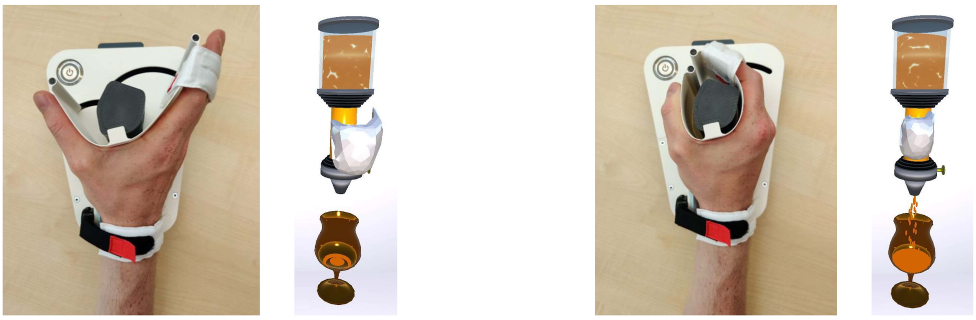

To see the next step, push the device button once quickly.

3 Game instructions

- To move from one bottle to other you have to open your hand at the Shell.
- Then rotate your forearm to the side you want move to.

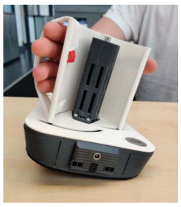
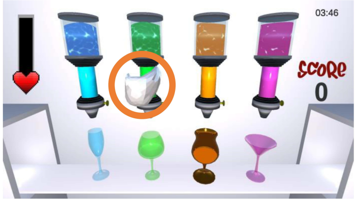

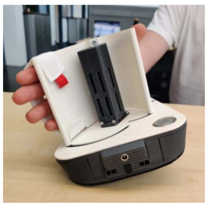
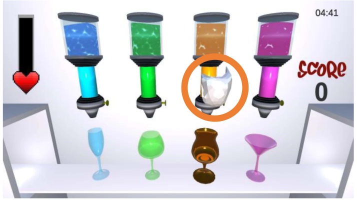

To see the next step, push the device button once quickly.

Emergency button use

- You can press the emergency stop button at any time without justification.

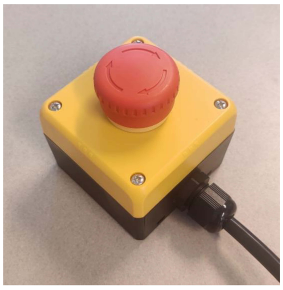

Important reminder

- Remember this is a simulated unsupervised environment.
- Try to solve all questions you have on your own.
- If you are absolutely certain you can't resolve the problem by your own, you can ask the researcher as a last resort.

To see the next step, push the device button once quickly.

Play the game

- Push the device button once quickly to start the game.

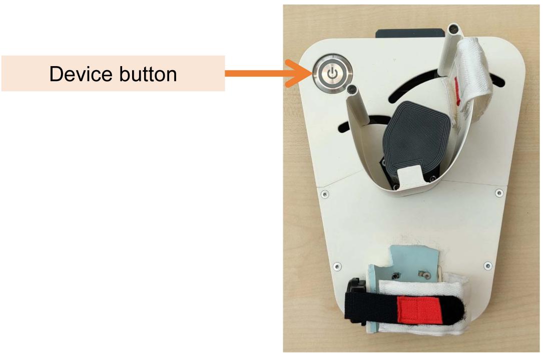

1 Turn off the device

- Press the button at least for 3 seconds to turn off the device.

2 Release hand

- Open the wrist strap.
- Optional: Open the finger strap.
- Release hand from the device.

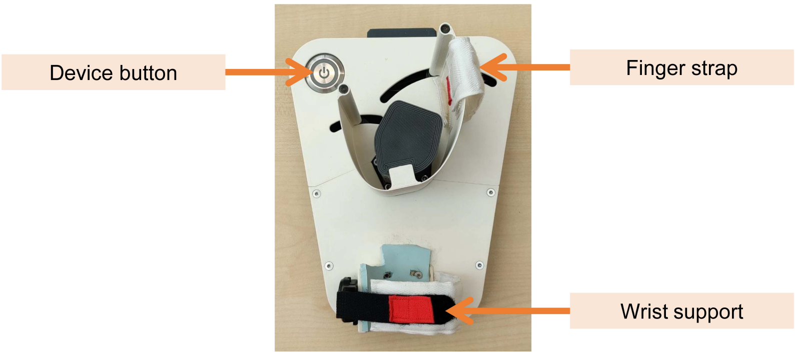

Supplement: Supplementary file 1 [file Data_Sheet_1.PDF]
